# Anagrelide Remodels the PDE3A–SLFN12 Interactome to Associate with Translation Initiation Machinery in Cancer Cell Lines

**DOI:** 10.1016/j.mcpro.2026.101663

**Published:** 2026-09-18

**Authors:** Sami Salmikangas, Iina Lindholm, Nasrin Sultana, Nanna Merikoski, Kirsi Toivanen, Xiaonan Liu, Markku Varjosalo, Tom Böhling, Tiina Vesterinen, Harri Sihto

**Affiliations:** 1 Rare Cancers Research Group, Department of Pathology, University of Helsinki and Helsinki University Hospital, Helsinki, Finland; 2 Stem Cells and Metabolism Research Program, Faculty of Medicine, University of Helsinki, Helsinki, Finland; 3 Gracile Research Group, Folkhälsan Research Center, Helsinki, Finland; 4 Institute of Biotechnology, Helsinki Institute of Life Science HiLIFE, University of Helsinki, Helsinki, Finland; 5 HUS Diagnostic Center, Department of Pathology, University of Helsinki and Helsinki University Hospital, Helsinki, Finland

**Keywords:** phosphodiesterase 3A, schlafen 12, molecular glues, interactomics, translation initiation

## Abstract

Phosphodiesterase 3A (PDE3A) modulators such as anagrelide induce complex formation between PDE3A and Schlafen 12 (SLFN12), selectively killing cancer cells that co-express both proteins. As PDE3A forms isoform- and cell type-specific signalosome complexes across multiple subcellular compartments, the effects of its modulation are expected to depend strongly on the surrounding protein interaction network. However, despite considerable preclinical and early clinical interest in PDE3A modulators, the cellular context in which these compounds act has remained poorly characterized. Using proximity-dependent biotinylation in two human cancer cell lines (SA-4, liposarcoma; HeLa, cervical adenocarcinoma), we mapped the interactomes of PDE3A, SLFN12, and the anagrelide-induced PDE3A–SLFN12 complex. Anagrelide induced 259 high-confidence interactions, most notably with ribosomal proteins and translation initiation factors, while suppressing 877 interactions, most prominently those associated with the proteasome, protein folding, and the ER membrane. Interactions were selectively induced with peripheral eukaryotic translation initiation factor 3 (eIF3) subunits eIF3A and eIF3B, while interactions with core eIF3 subunits eIF3H, eIF3L, and eIF3M were suppressed. These findings were validated by native co-immunoprecipitation across three cancer cell lines, and multiplex immunofluorescence confirmed accumulation and ribosomal redistribution of both PDE3A and SLFN12 following anagrelide treatment. AlphaFold modeling of the PDE3A–SLFN12 complex with a partial 43S pre-initiation complex predicted binding near eIF3B and the eIF2αβγ–tRNA ternary complex at the mRNA entry channel. By mapping the interactomes surrounding the drug-induced complex, our study provides important mechanistic context for how PDE3A modulators act within the cell. In our proposed model, anagrelide remodels the interactome of the PDE3A-SLFN12 complex, which relocalizes and accumulates near the mRNA entry channel of the 43S pre-initiation complex.

## Highlights

• Anagrelide remodels PDE3A and SLFN12 protein–protein interaction networks.
• Anagrelide induces PDE3A–SLFN12 complex accumulation and relocalization to ribosomes.
• The complex selectively interacts with peripheral eIF3 subunits eIF3A and eIF3B.
• Computational modeling places PDE3A–SLFN12 at the 40S mRNA entry channel.
• Proteasome and chaperonin interactions are broadly suppressed by anagrelide.

## Graphical Abstract

**Figure:**
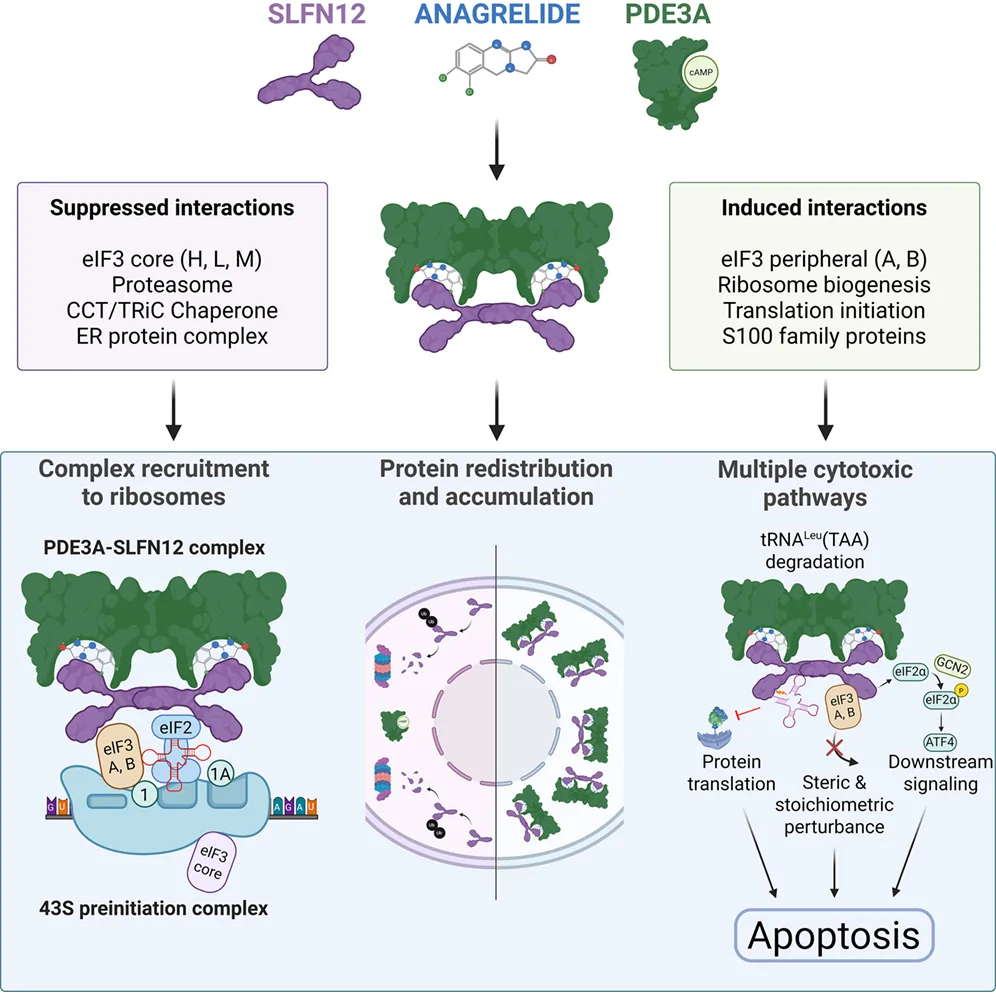

## In Brief

PDE3A modulators kill cancer cells by inducing complex formation between PDE3A and SLFN12, but the broader mechanistic and cellular context of this complex remains poorly understood. Using proximal biotinylation interactomics in human cancer cell lines, we show that anagrelide drives the PDE3A–SLFN12 complex to accumulate at cytoplasmic ribosomes, where it selectively interacts with peripheral eIF3 subunits at the mRNA entry channel of the 40S ribosomal subunit, while associations with the proteasome and chaperonin complexes are reduced.

Phosphodiesterases (PDEs) orchestrate the compartmentalization of cyclic nucleotide signaling. Cyclic adenosine monophosphate (cAMP) and cyclic guanosine monophosphate (cGMP) are ubiquitous second messengers, and more than 100 PDE isoforms are differentially localized to distinct subcellular compartments by specific anchoring proteins or localization motifs. PDEs can hydrolyze either cAMP or cGMP, or both, and the enzymes differ in regulation, structure, tissue expression, and subcellular localization, thereby providing specific control over cyclic nucleotide signaling within distinct tissues and organelles (1).

Phosphodiesterase 3A (PDE3A) is expressed as two transcript variants that give rise to three enzyme isoforms: PDE3A1, PDE3A2, and PDE3A3. All isoforms share an identical C-terminal catalytic domain, which is sufficient to confer sensitivity to PDE3A modulators (2), but differ in the length and composition of their N-terminal regulatory domain. The N-termini of PDE3A1 and PDE3A2 contain hydrophobic regions that direct the proteins to membrane structures of the endoplasmic reticulum (ER), particularly the perinuclear ER (3). In the absence of these regions, the PDE3A3 isoform localizes exclusively to the cytosol and, in some cases, the nucleus. The N-terminus also carries phosphorylation sites—three in PDE3A1 and two in PDE3A2—that modulate enzymatic activity and subcellular compartmentalization of the isoforms (4). Given that PDE3A isoforms interact with a variety of proteins in multiple subcellular compartments (reviewed in (5)), the functional consequences of PDE3A inhibition or modulation are expected to depend strongly on the composition of the surrounding signalosome.

Cytotoxic PDE3A modulators, also known as velcrins (6), are a small but structurally diverse class of molecular glues that promote complex formation between PDE3A and Schlafen-12 (SLFN12) (2, 6, 7, 8). The class includes compounds such as DNMDP (7), anagrelide (9), zardaverine (10), nauclefine (11), and 17-β-estradiol (E2) (12), which have been shown to kill cancer cells that co-express PDE3A and SLFN12. PDE3A expression level correlates with, and is proposed to determine, the cellular sensitivity to PDE3A modulators (12, 13), and multiple soft-tissue sarcomas, including gastrointestinal stromal tumor (GIST) (9, 14) and liposarcoma (15), have been reported to overexpress PDE3A. Some PDE3A modulators also inhibit PDE3A enzymatic activity, but their cytotoxicity does not depend on it, as PDE3A inhibitors without SLFN12 binding capabilities, such as trequinsin and cilostazol, are not cytotoxic to cancer cells (6, 7, 9).

Belonging to the intermediate-length subgroup of the Schlafen family, SLFN12 lacks a C-terminal helicase/ATPase domain and nuclear targeting sequence found in longer SLFNs, thus rendering it mainly cytosolic (16, 17, 18). SLFN12 is interferon-inducible and has been shown to inhibit HIV-1 latency reversal with a codon usage-dependent transcriptional block, mediated by its selective cleavage of tRNA^Leu^(TAA)—a rare tRNA codon in human transcripts but one enriched in HIV-1 and HIV-2 sequences (19). Under normal conditions, SLFN12 is a fast-turnover protein with a half-life of less than 2 hours (20), and its abundance is maintained at low levels via constitutive ubiquitin-mediated protein degradation (12). Heterodimerization of the PDE3A–SLFN12 complex, induced by PDE3A modulators, prevents SLFN12 degradation and activates its cytotoxic RNase function (12). This leads to cellular tRNA^Leu^(TAA) cleavage, halting of protein translation, activation of the integrated stress response (ISR), and, ultimately, cell cycle arrest and cell death (21, 22). However, as the subcellular compartmentalization and protein–protein interactions (PPIs) of PDE3A vary depending on the protein isoform, cell type, phosphorylation status, and a variety of other parameters (5), the effect of PDE3A modulation is expected to be cell type- and compartment-specific. Yet, the cellular compartments, PPIs, and surrounding signalosomes of the modulator induced PDE3A-SLFN12 complex are largely unknown. A broader mechanistic understanding of how PDE3A modulators redirect PDE3A, SLFN12, and/or the PDE3A-SLFN12 complex within the cell is essential for rational optimization of PDE3A modulation-based therapeutics.

Here, we used BioID, a proximity-dependent biotinylation approach (23), to map the proximal interactomes of PDE3A, SLFN12, and an anagrelide-induced PDE3A–SLFN12 complex in SA-4 (liposarcoma) and HeLa (cervical adenocarcinoma) cell lines. Anagrelide treatment increased interactions with ribosomal proteins and peripheral eukaryotic initiation factor 3 (eIF3) subunits, while interactions with core eIF3, proteasome, and chaperonin complexes were suppressed. By combining the interactomic screening with multiplex immunofluorescence and AlphaFold computational structural modeling, our results show that the anagrelide-induced PDE3A-SLFN12 complex accumulates and relocalizes to the ribosomal mRNA entry channel and interacts with the translation initiation machinery.

## Experimental Procedures

### Experimental Design and Statistical Rationale

#### Cloning

Protein expression vectors were generated by cloning the genes of interest (PDE3A isoform 1 (PDE3A1), RefSeq NM_000921.5; PDE3A isoform 2 (PDE3A2), RefSeq NM_001244683.2; or SLFN12, RefSeq NM_018042.5) into the MAC-tag-N plasmid backbone (24) (Cat. No. 108078, Addgene; https://www.addgene.org/browse/sequence/209206/), where the biotin ligase is embedded in the N-terminus of the bait protein. A single N-terminal BioID fusion was used rather than a combination of N-terminal and C-terminal fusions because a C-terminal biotin ligase was found to interfere with PDE3A-SLFN12 complex formation. The MAC-tag-GFP construct used for control samples for BioID was kindly provided by Dr Markku Varjosalo (Institute of Biotechnology, University of Helsinki).

Cloning was carried out according to the manufacturer’s instructions using Gateway BP Clonase II Enzyme mix (Cat. No. 11789020, Invitrogen, Thermo Fisher Scientific) and Gateway LR Clonase II Enzyme mix (Cat. No. 11791020, Invitrogen). Primer sequences used for generating the attB-flanked PCR products were: SLFN12-FOR: *GGGG ACA AGT TTG TAC AAA AAA GCA GGC TCC ACC ATG AAC ATC AGT GTT GAT TTG*; SLFN12-REV: *GGG GAC CAC TTT GTA CAA GAA AGC TGG GTC CTA GGT GAG CCT TCG ACA AGA*; PDE3A1-FOR: *GGGG ACA AGT TTG TAC AAA AAA GCA GGC TCC ACC ATG GGC TTG TAC CTC CTG CGC*; PDE3A2-FOR: *GGGG ACA AGT TTG TAC AAA AAA GCA GGC TCC ACC ATG TCC GGC TGC AGC AGC AAG*; and PDE3A-REV: *GGG GAC CAC TTT GTA CAA GAA AGC TGG GTC TCA CTG GTC TGG CTT TTG GGT*. Stop codons were included at the 3′ ends of the coding sequences to prevent translation of additional C-terminal residues that could interfere with the interaction between the catalytic domain of PDE3A (PDE3A^CAT^) and the C-terminal PDE3A interacting region (PIR) of SLFN12. PCR was performed using Platinum SuperFi PCR Master Mix (Cat. No. 12358-050, Invitrogen) according to the manufacturer’s instructions. The PCR cycling conditions were initial denaturation at 98 °C for 2 min, followed by 30 cycles of denaturation at 98 °C for 10 s, annealing at 67 °C for 10 s, and elongation at 72 °C for 60 s and a final elongation at 72 °C for 5 min.

#### Cell Culture

GIST882 (RRID:CVCL_7044) cell line was kindly provided by Dr Jonathan Fletcher (Harvard Medical School); SA-4 (RRID:CVCL_8910) cell line was kindly provided by Dr Kjetil Boye (Oslo University Hospital); and the HeLa (RRID:CVCL_0030) cell line was kindly provided by Professor Leif Andersson (University of Helsinki).

Cell lines were authenticated using short-tandem-repeat profiling at the Institute for Molecular Medicine Finland (FIMM) Genomics Unit (HiLIFE infrastructures, University of Helsinki and Biocenter Finland). GIST882 was verified to contain a known *KIT* exon 13 p.K642E mutation by Sanger sequencing. All cell lines had been regularly tested negative for *mycoplasma* and were cultured in a humidified, 5% CO_2_ atmosphere at 37 °C. GIST882 and SA-4 were cultured in RPMI 1640 (Cat. No. 31870025, Gibco) and HeLa was cultured in DMEM (Cat. No. 21969035, Gibco), with added 1X Pen Strep Glut (Cat. No. 10378016, Gibco) and 10% (HeLa and SA-4) or 20% (GIST882) FBS (Cat. No. A5256801, Gibco).

#### BioID

BioID was performed as previously described (25) in SA-4, HeLa, and GIST882 cell lines, except the generation of cell lines stably expressing the MAC-tagged protein of interest was replaced with a transient transfection with the respective constructs (MAC-PDE3A1-N, MAC-PDE3A2-N, MAC-SLFN12-N, or MAC-GFP-N). In GIST882, however, the assay did not yield sufficient results due to transfection inefficiency. Briefly, cells were grown to 70% confluence (3 x T175 flasks per replicate) and transfected with a 1:1 (m/v) ratio of plasmid DNA to jetOPTIMUS transfection reagent (Cat. No. 101000006, Polyplus, Sartorius, Illkirch, France) according to the manufacturer’s instructions. After 24 h, the transfected cells were treated with fresh-made 50 μM D-Biotin (Cat. No. BP232-1, Fisher Bioreagents, Thermo Fisher Scientific) and 100 nM anagrelide (approximate IC50 value for SA-4 and GIST882 (15); Cat. No. 735554, Lancrix), or 50 μM biotin and 1% DMSO (vehicle control). Two biological replicates of each sample and four biological replicates of the GFP-control were prepared. After 24 h of treatment, the cells were pelleted and flash-frozen in liquid nitrogen. Protein purification, liquid chromatography–mass spectrometry, and protein identification was performed as described elsewhere (25) at the Institute of Biotechnology, Proteomics Unit (University of Helsinki). Briefly, cell pellets were lysed in BioID lysis buffer, sonicated, and treated with benzonase. Affinity purification was done using Strep-Tactin beads, after which bound proteins were eluted with 0.5 mM D-biotin, reduced with 5 mM TCEP, alkylated with 10 mM iodoacetamide, and digested with sequencing grade trypsin. Desalted peptides were analyzed by LC-MS/MS using an Evosep One system coupled to a Bruker timsTOF Pro/Pro 2 mass spectrometer. Separation was performed on a 150 μm × 8 cm C18 column over a 21-min gradient (60 samples per day method). Data were acquired in positive-ion, data-dependent acquisition (DDA) mode using Parallel Accumulation Serial Fragmentation (PASEF). All raw data files were processed and searched using FragPipe software suite with the MSFragger search engine (26) in one batch per cell line (SA-4 cell line batch acquisition date June 2023, FragPipe v19.1, MSFragger 3.7 and Philosopher 4.8.0; HeLa cell line batch acquisition date January 2024, FragPipe v21.1, MSFragger 4.0 and Philosopher 5.1.0). Spectra were searched against the human UniProtKB protein sequence database (taxonomy 9606; the 2023-06-16 release comprising 20,426 entries for SA-4, and the 2024-01-24 release comprising 20,434 entries for HeLa), to which the MAC-tag and GFP sequences, common contaminants, and reversed-sequence decoys were appended. Trypsin was specified as fully tryptic with up to two missed cleavages, and precursor and fragment mass tolerances were each ±20 ppm. Carbamidomethylation of cysteine was set as a fixed modification, while variable modifications comprised N-terminal acetylation, methionine oxidation, and biotinylation of lysine residues and peptide N-termini. For the SA-4 batch, peptide-spectrum matches were validated with PeptideProphet; for the HeLa batch, MSBooster rescoring and Percolator were used, followed by iProphet. Protein inference was performed with ProteinProphet in both batches, and results were filtered with the Philosopher (27) tool to a false discovery rate below 1% at the peptide-spectrum match, peptide, and protein levels. Interactor abundance was quantified by spectral counting. All proteins identified in each cell line batch, with their distinct peptide counts, sequence coverage, and per-run spectral counts, are listed in Supplemental Table S4.

High-confidence interactors were identified using the Significance Analysis of INTeractome (SAINT) (28). For each bait, MAC-GFP runs serve as the negative-control counts in SAINT, which assigns each bait–prey pair a probability of being a true interaction versus background, yielding a Bayesian FDR (BFDR). We retained interactions with ≥10 average spectral count, ≥5% average spectral count of bait protein abundance, and a Bayesian false discovery rate (BFDR) of ≤0.05. Additionally, we used the CRAPome (29) database to exclude common contaminant proteins present in ≥20% of CRAPome control samples, unless their average spectral count fold change was ≥3. Protein–protein interaction data was visualized using a dotplot generated via the ProHits-viz online tool (https://prohits-viz.org/).

#### Interaction Mapping

Spectral counts of individual proteins were normalized to each sample’s bait protein abundance, and bait-normalized average spectral count fold changes were compared between treatments to obtain a list of induced and suppressed protein-protein interactions. The lists were built into interaction networks using the STRING database version 12.0 (https://string-db.org/) (30), clustered with MCL clustering using an inflation value of 3 and visualized with Cytoscape version 3.10.2 (31). The Gene Ontology (GO) term with the best signal (defined as a weighted harmonic mean between the observed/expected ratio and -log(FDR)) in the functional enrichments analysis in STRING was used as the description for each cluster.

#### Immunofluorescence

HeLa and SA-4 cells were seeded onto 12-well plates containing glass coverslips at a density of 2 × 10^5^ cells per well. After 24 h, cells were treated with 100 nM anagrelide or 1% DMSO (vehicle control). After 24 h of treatment, the cells were fixed with 4% paraformaldehyde, permeabilized with 0.4% Triton X-100, and blocked with 2.5% BSA in PBS. Cells were incubated overnight at 4°C with primary antibodies against mouse PDE3A (Antibody Ab 230-F8-G1, 1:4000, Sartar Therapeutics, Helsinki, Finland (14)) and rabbit SLFN12 (Cat. No. ab234418, 1:200, Abcam). After washing, cells were incubated overnight at 4 °C with secondary antibodies Alexa Fluor 633 anti-mouse (Cat. No. A21052, 1:1000, Invitrogen) and Alexa Fluor 750 anti-rabbit (Cat. No. A21039, 1:1000, Invitrogen). Finally, cells were incubated with Alexa Fluor 488 anti-RPS20 (Cat. No. ab309883, 1:400, Abcam) for 2 h at room temperature and nuclei were counterstained with Hoechst 33,342 (Cat. No. H3569, 1 μg/ml in PBS, Thermo Fisher Scientific) for 15 min at room temperature.

Imaging was performed using a Leica Stellaris 8 FALCON/DLS confocal microscope on an inverted Leica DMi8 microscope, controlled by LAS X software (Leica Microsystems). A 63X/1.40 NA oil immersion objective was used for imaging. Images were acquired with HyD hybrid detectors. Hoechst was visualized using a 405 nm laser for excitation and an emission window of 430 to 470 nm; RPS20 was visualized using a 488 nm laser for excitation and an emission window of 520 to 560 nm; PDE3A was visualized using a 638 nm laser for excitation and an emission window of 680 to 740 nm; and SLFN12 was visualized using a 730 nm laser for excitation and an emission window of 800 to 880 nm.

#### Image Analysis

Accumulation analysis was performed on sum slices Z-projections of confocal microscopy images using ImageJ (NIH). Regions of interest (ROIs) were manually drawn around individual cells, along with a background ROI lacking specific signal. Raw integrated density was measured for the PDE3A and SLFN12 channels, and mean background intensity multiplied by the cell area was subtracted from each cell’s total IF intensity. In total, 71 DMSO-treated HeLa cells, 75 ANA-treated HeLa cells, 50 DMSO-treated SA-4 cells, and 38 ANA-treated SA-4 cells were analyzed for total IF intensity. Colocalization analysis was performed using the Coloc2 plugin in ImageJ, for which 55 HeLa cells and 69 SA-4 cells per treatment group were analyzed, and colocalization quantified as Pearson’s correlation coefficients (32). For both analyses, data were assessed for normality using the Shapiro–Wilk test, and statistical comparisons between treatment groups were performed using the nonparametric Mann–Whitney *U* test. Statistical analyses were carried out in GraphPad Prism version 10.1.2, and *p*-values of less than 0.05 were considered significant.

For representative images, maximum intensity Z-projections were used. Minimum and maximum display ranges for each fluorescence channel were fixed across all samples and applied identically to treated and control images. No nonlinear scaling or gamma correction was applied.

#### Co-IP and Western Blotting

Co-IP was performed using Pierce Crosslink Immunoprecipitation Kit (Cat. No. 26147, Invitrogen) according to the manufacturer’s instructions, using 10 μg of PDE3A antibody (Cat. No. A302-740A, Bethyl Laboratories) per reaction. All cell samples were native non-transfected and treated with 100 nM anagrelide or 1% DMSO for 24 h before lysis. Input sample (IN), unbound flowthrough (FT), and eluted sample (IP) were run on 10% Mini-PROTEAN TGX Gels (Cat. No. 4561036, Bio-Rad), blotted using Trans-Blot Turbo Transfer Packs (Cat. No. 1704156, Bio-Rad), blocked with EveryBlot Blocking Buffer (Cat. No. 12010020, Bio-Rad), and probed with the following antibodies: PDE3A 1:1000 (Cat. No. HPA014492, Sigma-Aldrich), SLFN12 1:500 (Cat. No. ab234418, Abcam), eIF3A 1:1000 (Cat. No. 27665-1-AP, Proteintech), eIF3B 1:1000 (Cat. No. 10319-1-AP, Proteintech), eIF3G 1:1000 (Cat. No. 11165-1-AP, Proteintech), eIF3H 1:1000 (Cat. No. 11310-1-AP, Proteintech), eIF3I 1:1000 (Cat. No. 11287-1-AP, Proteintech), eIF3L 1:1000 (Cat. No. 11482-1-AP, Proteintech), eIF3M 1:1000 (Cat. No. 11423-1-AP, Proteintech), and actin 1:100,000 (Cat. No. A300-491A, Bethyl Laboratories). A goat-anti-rabbit secondary antibody 1:10,000 (Cat. No. 111-035-003, Jackson ImmunoResearch) was used for all primary antibodies, and SuperSignal West Pico PLUS Chemiluminescent Substrate (Cat. No. 34580, Thermo Fisher Scientific) was used for detection. The membranes were imaged using the Azure 500 imaging system (Azure Biosystems). For densitometric analyses, band intensities were quantified manually in ImageJ from lane profile plots. Each peak was separated from the local background at its base, and the area of the peak was taken as the band intensity.

Small-interfering RNA (siRNA) assays were used to validate the Co-IP results for eIF3G and eIF3I using pre-designed siRNA sets (Cat. No. HY-RS04263 for eIF3G and Cat. No. HY-RS04265 for eIF3I, MedChemExpress), and Lipofectamine RNAiMAX (Cat. No. 13778075, Invitrogen) for transfection. The siRNA assays were performed according to the manufacturer’s instructions, while Co-IP and Western blotting for the siRNA samples were performed as above.

#### Computational Modeling of PDE3A–SLFN12 Complex

The 3D structure of the PDE3A–SLFN12 complex interacting with the translation initiation machinery was modeled using AlphaFold Server (33). Two PDE3A catalytic domains (residues 669-1141, NP_000912.3) and two full-length SLFN12 proteins (NP_060512.3) were modeled together with a single copy of tRNA-Met (HG983954.1), eIF2α (NP_004085.1), eIF2γ (NP_001406.1), eIF3A C-terminal domain (NP_003741.1, residues 573-706), eIF3B (NP_001032360.1), eIF3G (NP_003746.2), and eIF3I (NP_003748.1) molecules. Molecular graphics for the resulting structure model were performed with UCSF ChimeraX version 1.8 (34).

## Results

### Anagrelide Induces PDE3A–SLFN12 Complex Interaction with Translation Initiation Machinery

We performed a BioID proximal interactome analysis using an N-terminal promiscuous biotin ligase (BirA∗) fused to a full-length PDE3A1, PDE3A2, or SLFN12 as bait proteins in two distinctly differing PDE3A modulator-sensitive cell lines (HeLa – cervical adenocarcinoma, epithelial; and SA-4 – liposarcoma, mesenchymal) with a 24-h anagrelide treatment or vehicle control (DMSO). MAC-GFP was transfected and expressed in parallel in every condition and served as the control bait. Transfected PDE3A1 and PDE3A2 accumulated to 1.5 to 2.9-fold over the endogenous protein and transfected SLFN12 to higher levels (Supplemental Fig. S1), and replicate spectral counts correlated well (Pearson r = 0.816–0.916, Supplemental Fig. S2*A*), with differences between the 2 cell lines exceeding within-replicate variability 2.2- to 4.7-fold (Supplemental Fig. S2*B*). In total, 4141 proteins were identified in the SA-4 batch and 3988 in the HeLa batch, of which 4921 unique preys were scored by SAINT (Supplemental Table S4 and Fig. 1*B*). After removing low-abundance, low-confidence, and common-contaminant interactors, a total of 562 high-confidence interactors were identified across all cell lines and bait proteins (Fig. 1 and Supplemental Table S1).

**Fig. 1:**
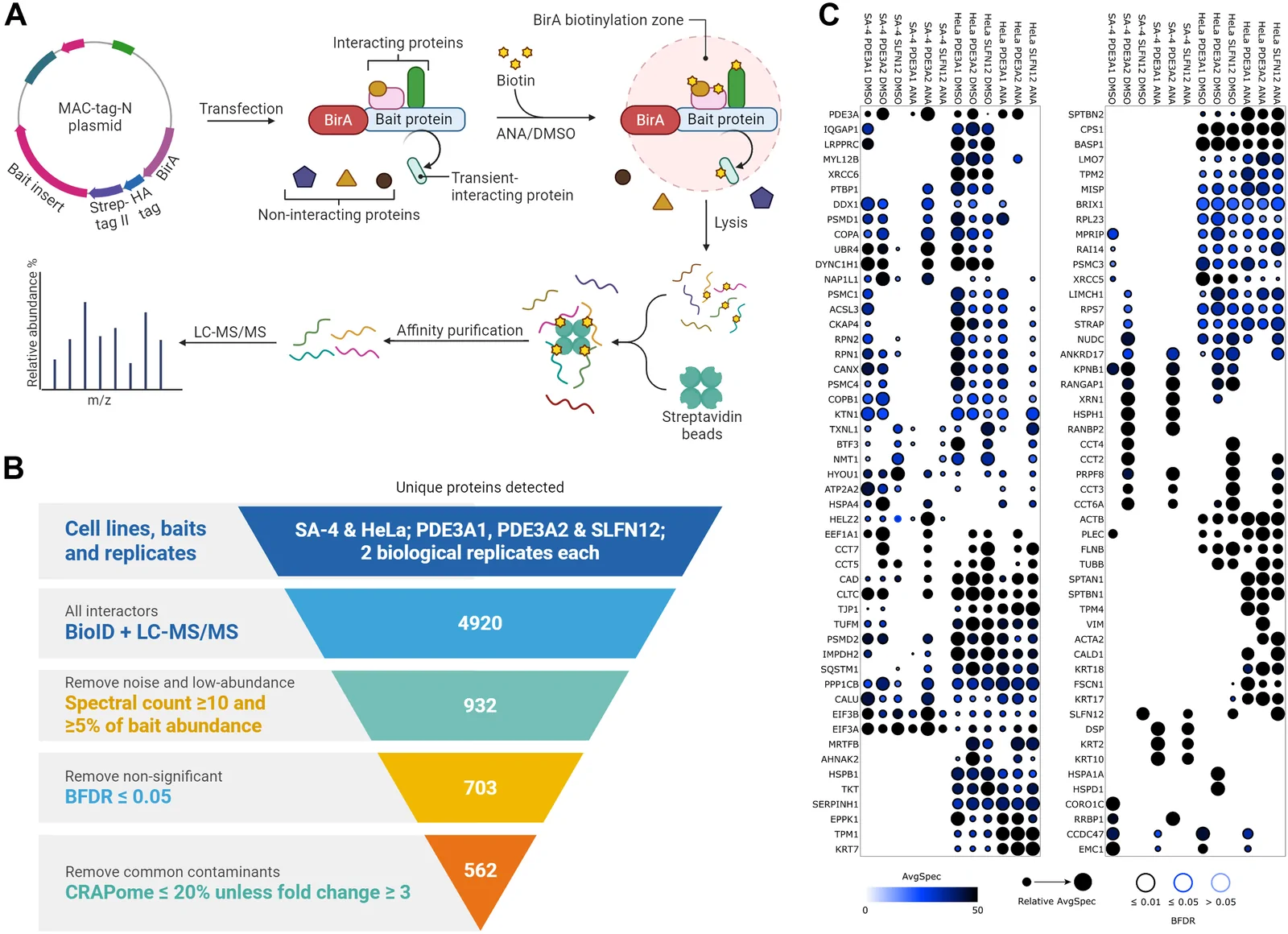
**BioID workflow, filtering pipeline, and high-confidence proximal interactors of PDE3A and SLFN12.***A*, experimental design and workflow. PDE3A1, PDE3A2, SLFN12 and GFP were expressed as N-terminal MAC-tag fusions carrying the promiscuous biotin ligase BirA. After transient transfection and biotin supplementation, proteins within the labeling radius of the bait are biotinylated, affinity purified on streptavidin beads following lysis, and identified by LC-MS/MS. *B*, high-confidence interactor filtering pipeline, showing the number of unique proteins retained at each step. Samples comprised 2 cell lines (SA-4 and HeLa), three baits and two biological replicates each. High-confidence interactors were required to have an average spectral count of ≥10 and ≥ 5% of bait abundance, a BFDR of ≤0.05 in SAINT against the MAC-GFP controls, and to be present in ≤20% of CRAPome control experiments unless their average spectral count fold change was ≥3, yielding 562 high-confidence interactors. *C*, Dot plot of the 100 most abundant high-confidence interactors across all conditions. *Dot* size indicates the average spectral count relative to the highest value observed for that protein across the 12 conditions, fill color the absolute average spectral count, and outline color the BFDR. *Created in BioRender*. *Salmikangas*, *S*. (2026) https://BioRender.com/*k0lp9oh*.

Among the 562 high-confidence interactors, anagrelide induced 259 bait–prey interactions (newly detected or increased in relative abundance) and suppressed 877 (lost or decreased) (Supplemental Table S1). We mapped these interactions into clustered interaction networks using the STRING database (30) (Fig. 2 and Supplemental Table S2). Induced interactions consisted of multiple networks involved in translation such as ribosomal proteins, ribosome biogenesis factors, and eukaryotic translation initiation factors (eIFs), while suppressed interactions were most notably associated with protein folding, the proteasome complex, and translation (Fig. 2). Of the 562 interactors, 105 were exclusively induced, 391 were exclusively suppressed, and 66 showed bait-specific directionality (induced interactions with one bait but suppressed with another). Interactions induced in both cell lines were limited to translation initiation-related proteins, whereas networks suppressed in both cell lines included components of the translation, proteasome, chaperonin, and ER–protein complexes (Fig. 2*C*).

**Fig. 2:**
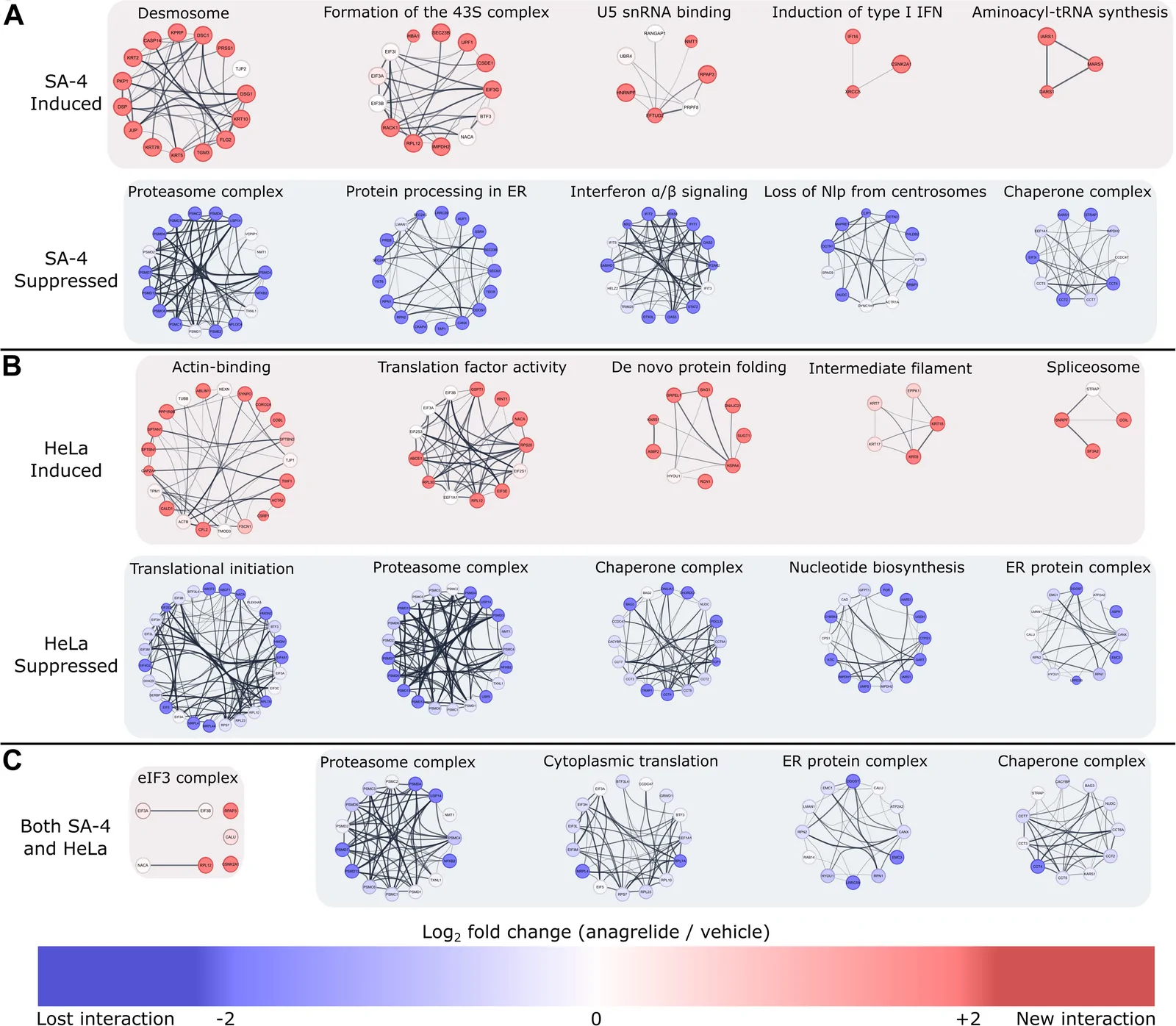
**Clustered networks of anagrelide-modulated protein-protein interactions.** High-confidence interactors of the three baits (PDE3A1, PDE3A2 and SLFN12) were combined per cell line, and anagrelide-treated samples were compared with vehicle-treated samples. Interactions induced and suppressed by anagrelide are shown in (*A*) SA-4 cells, (*B*) HeLa cells, and (*C*) both cell lines. Networks were clustered by MCL in the STRING database version 12.0 with an inflation parameter of 3, and each cluster was named after the Gene Ontology term with the best signal in the STRING functional enrichment analysis. The five largest clusters are shown per category; all clusters and their members are listed in Supplemental Table S2. Node fill indicates the log2 fold change in bait-normalized average spectral count between anagrelide and vehicle, taken as the highest value across the three baits. All observed fold changes fell within ±2, and the darker segments at each end of the scale denote interactions detected in only one treatment, which cannot be expressed as a ratio: lost (detected in vehicle only) and new (detected in anagrelide only).

In addition to translation-related proteins, induced interactions included multiple desmosome-related proteins in SA-4 cells (Fig. 2*A*) and actin-binding proteins in HeLa cells (Fig. 2*B*). Subunits of several large multiprotein complexes, including the proteasome regulatory 19S particle (PSMCs and PSMDs), chaperonin containing TCP-1 complex (CCTs), and endoplasmic reticulum membrane complex (EMCs), were broadly suppressed across both cell lines. Interactors related to interferon signaling, including 2′-5′-oligoadenylate synthetases OAS2 and OAS3 associated with antiviral responses, were suppressed specifically in SA-4 cells (Fig. 2*A*).

The eIF3 multiprotein complex is of particular interest, as interactions with specific eIF3 subunits were either induced (subunits A and B) or suppressed (subunits H, L, and M) consistently in both cell lines (Fig. 2*C* and Supplemental Table S1). The mammalian eIF3 complex consists of 13 subunits organized into two major modules. The octamer core, comprising subunits A, C, E, F, H, K, L, and M, is positioned at the mRNA exit site of the 40S ribosomal subunit, whereas the peripheral module, composed of subunits B, G, I, and the C-terminal domain of subunit A (eIF3A^CTD^), is located at the mRNA entry site (35). Notably, both subunits showing induced interactions with the PDE3A-SLFN12 complex in the BioID analysis are components of the peripheral module, while the subunits with suppressed interactions belong to the octamer core.

### PDE3A–SLFN12 Complex Interacts with eIF3A and eIF3B

Next, to determine whether the translation initiation machinery directly interacts with the PDE3A–SLFN12 complex or is merely in close enough proximity to become labeled in BioID, we performed native (non-transfected) PDE3A co-immunoprecipitation in SA-4, GIST882, and HeLa cells, treated with either DMSO or anagrelide, and probed for eIF3 subunits showing the strongest drug-dependent interaction changes: A, B, G, I, H, L, and M (Fig. 3*A*). GIST882 cells, which have previously been shown to be highly sensitive to anagrelide (9), were included as a third PDE3A-expressing model.

**Fig. 3:**
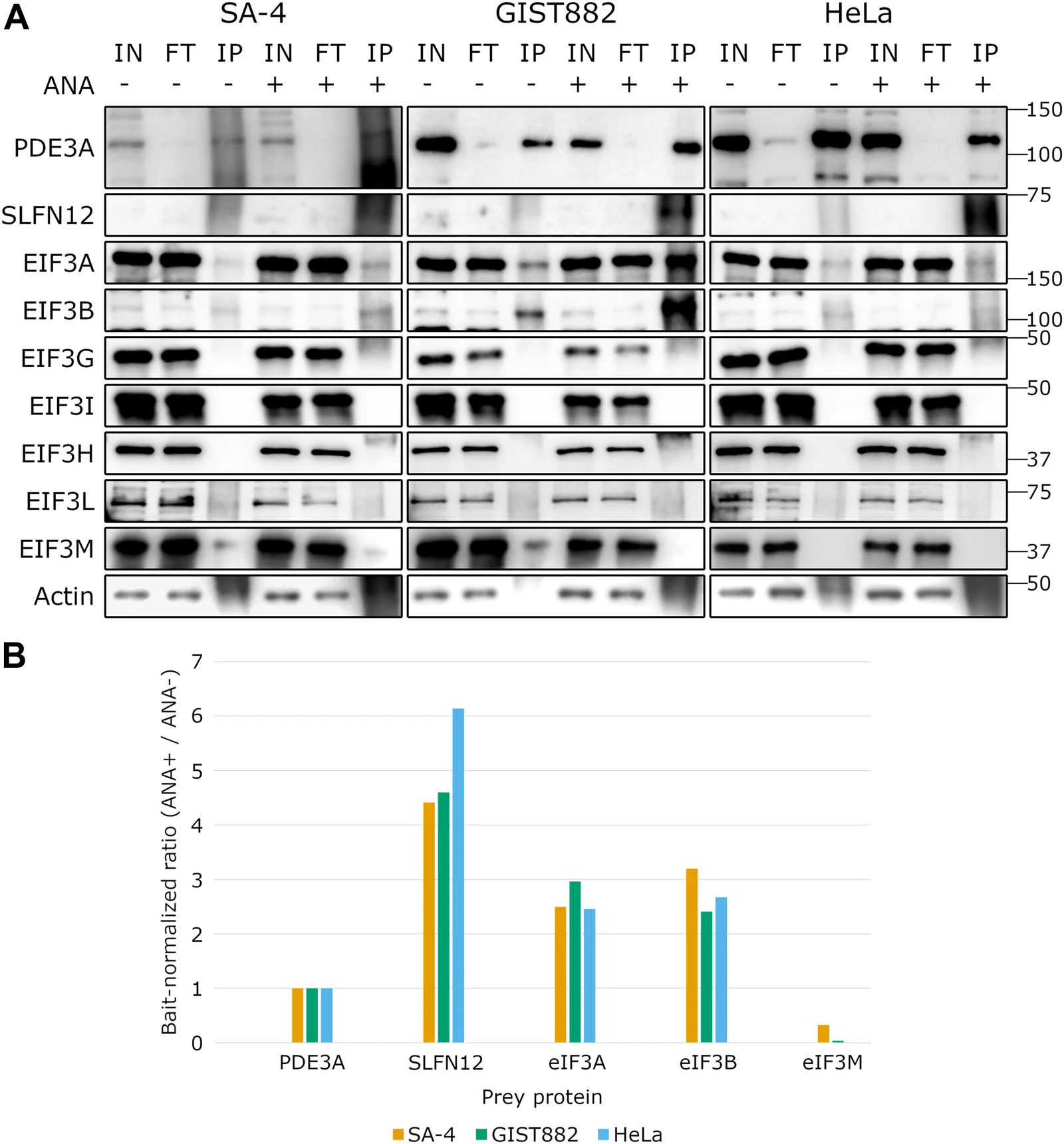
**Co-immunoprecipitation of eIF3 subunits with PDE3A in response to anagrelide treatment.***A*, SA-4, GIST882, and HeLa cells were treated with 1% DMSO (ANA-) or 100 nM anagrelide (ANA+) for 24 h. Native lysates were subjected to co-immunoprecipitation using a PDE3A antibody-conjugated resin. Input (IN), flowthrough (FT), and immunoprecipitated (IP) fractions were analyzed by immunoblotting for PDE3A, SLFN12 and the indicated eIF3 subunits. Actin was included as loading and specificity control. Molecular weight markers are given in kDa. *B*, densitometric quantification of the IP fractions shown in (*A*). Each prey band was normalized to the PDE3A bait band in the same lane, and the bait-normalized signal in anagrelide-treated samples was then expressed relative to the corresponding vehicle-treated sample. PDE3A equals 1 by definition and is shown for reference. The eIF3 subunits that gave no detectable IP signal in either condition (eIF3G, eIF3H, eIF3I and eIF3L) were not quantified.

Among the eIF3 subunits forming the peripheral module, eIF3A and eIF3B showed increased interaction with PDE3A following anagrelide treatment (Fig. 3*B*), whereas eIF3G and eIF3I did not co-immunoprecipitate (co-IP) with PDE3A (Fig. 3*A*). The additional band seen at 50 kDa in eIF3G co-IP samples was confirmed to be non-specific (Supplemental Fig. S3). In contrast, among the octamer core subunits, eIF3H and eIF3L did not interact with PDE3A under any condition, while eIF3M displayed decreased interaction in SA-4 and GIST882 cells after anagrelide treatment (Fig. 3*B*).

These results are consistent with the BioID proximal interactome analysis, in which interactions with eIF3A and eIF3B were consistently induced across most bait proteins and both cell lines, and interactions with eIF3C, eIF3E, eIF3G, and eIF3I showed variable or sample-specific interactions (Fig. 2*C* and Supplemental Table S1). In contrast, eIF3H, eIF3L, and eIF3M interactions were broadly suppressed while eIF3D, eIF3F, eIF3J, and eIF3K interactions were undetectable regardless of cell line or bait protein. Together, these data demonstrate that the PDE3A-SLFN12 complex interacts selectively with the peripheral module rather than the octamer core of eIF3, suggesting that the active complex localizes near the mRNA entry site of the 40S ribosomal subunit.

### PDE3A-SLFN12 Complex Relocalizes to Ribosomes

BioID results suggested that formation of the PDE3A–SLFN12 complex upon anagrelide treatment may shift the subcellular localization of both proteins toward cytoplasmic ribosomes, as indicated by increased interactions with ribosomal proteins and reduced interactions with ER- and nuclear-associated proteins (Fig. 2). To determine whether the PDE3A–SLFN12 complex associates directly with the translation initiation machinery at the ribosome, we performed multiplex immunofluorescence (IF) labeling of the nuclei, PDE3A, ribosomal protein S20 (RPS20), and SLFN12 (Fig. 4).

**Fig. 4:**
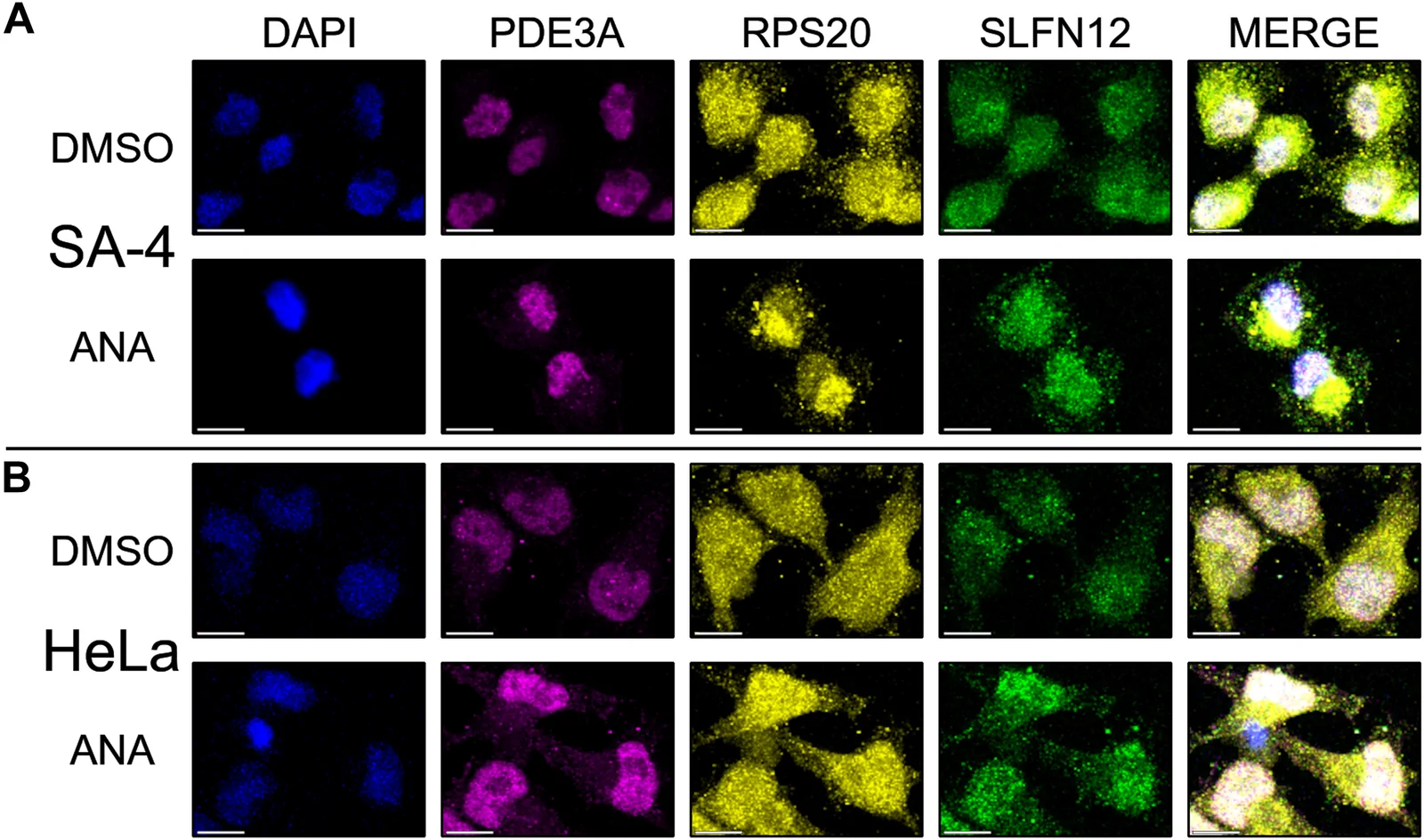
**Representative images of multiplex immunofluorescence in DMSO- or anagrelide-treated cells.***A*, SA-4 cells and (*B*) HeLa cells were treated with either 1% DMSO (DMSO) or 100 nM anagrelide (ANA) for 24 h and stained for nuclei (DAPI–*blue*), PDE3A (PDE3A–*magenta*), ribosomes (RPS20–*yellow*) and SLFN12 (SLFN12–*green*). In the overlay image (MERGE), colocalized PDE3A and SLFN12 appear as white pixels. Scale bar in each image is 10 μM.

Under control conditions, PDE3A localized predominantly to the perinuclear endoplasmic reticulum, consistent with previous reports (3), whereas SLFN12 and RPS20 displayed diffuse cytoplasmic distributions. Following anagrelide treatment, both PDE3A and SLFN12 displayed markedly increased and more punctate cytoplasmic and perinuclear ER staining, consistent with the idea of stabilization of the drug-induced complex and reduced turnover via the proteasomal pathway. We then quantified the extent of PDE3A and SLFN12 accumulation by measuring total IF intensity per cell (Fig. 5*A*) and redistribution toward ribosomes by measuring pairwise colocalization (Fig. 5*B*).

**Fig. 5:**
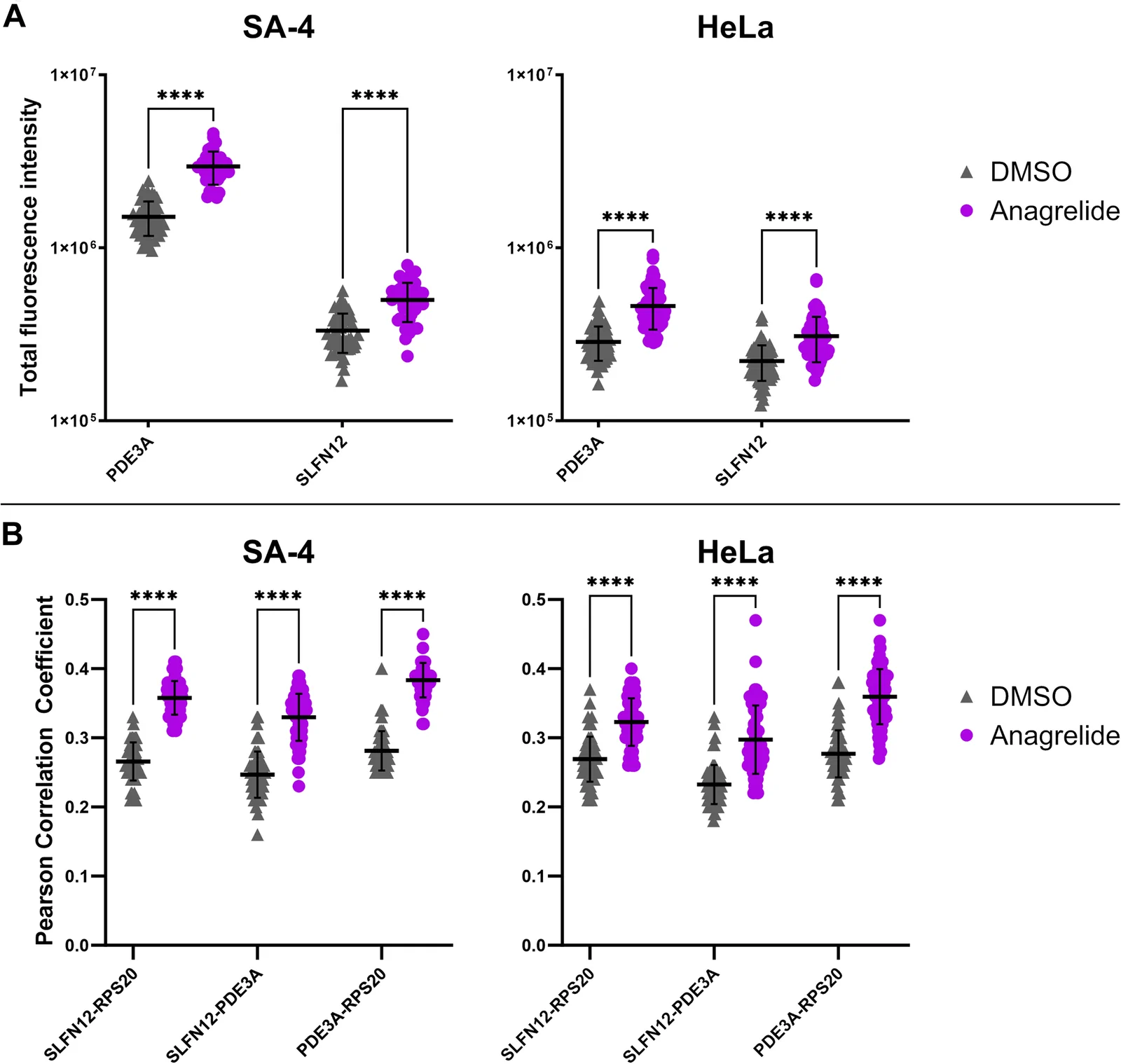
**PDE3A and SLFN12 accumulation and colocalization in response to anagrelide treatment.***A*, total immunofluorescence intensity of PDE3A and SLFN12 per cell in SA-4 and HeLa cells treated with 1% DMSO (*gray triangles*) or 100 nM anagrelide (*purple circles*). Each point represents 1 cell. Sample sizes: HeLa DMSO n = 71, HeLa anagrelide n = 75, SA-4 DMSO n = 50, SA-4 anagrelide n = 38. Data are plotted on a logarithmic (log10) y-axis for visualization. *B*, Pearson correlation coefficients for colocalization of SLFN12–RPS20, SLFN12–PDE3A and PDE3A–RPS20, in SA-4 and HeLa cells treated with 1% DMSO or 100 nM anagrelide. Sample sizes: HeLa DMSO n = 55, HeLa anagrelide n = 55, SA-4 DMSO n = 69, SA-4 anagrelide n = 69. Horizontal lines represent mean ± SD. ∗∗∗∗ = *p* < 0.0001, Mann-Whitney U-test.

Total IF intensity of PDE3A and SLFN12 per cell was significantly increased following anagrelide treatment in both SA-4 and HeLa cell lines (Fig. 5*A*), showing accumulation of both proteins. Mean increases in Pearson’s correlation coefficient for SLFN12–RPS20 (SA-4 Δ = 0.092, HeLa Δ = 0.041), SLFN12-PDE3A (SA-4 Δ = 0.083, HeLa Δ = 0.056), and PDE3A–RPS20 (SA-4 Δ = 0.102, HeLa Δ = 0.078) indicate a significant shift of PDE3A and SLFN12 colocalization toward cytoplasmic ribosomes and each other (Fig. 5*B* and Supplemental Table S3). Together with the BioID and co-immunoprecipitation data, these results support a model in which the anagrelide-induced PDE3A–SLFN12 complex accumulates at cytoplasmic ribosomes and interacts with specific subunits of the translation initiation machinery.

### AlphaFold Predicts PDE3A–SLFN12 Interaction with eIF3 Complex

To further characterize how the PDE3A–SLFN12 complex binds and interacts with the peripheral eIF3 module, we used AlphaFold Server to model the PDE3A–SLFN12 heterotetramer complex together with a partial 43S pre-initiation complex (43S PIC), including spatially closely located eIF2α, eIF2γ, eIF3A^CTD^, eIF3B, eIF3G, eIF3I, and tRNA-Met (Fig. 6). The model displays reasonably accurate structural predictions (Supplemental Fig. S4) of both the 43S PIC (Fig. 6*A*) and the PDE3A–SLFN12 heterodimer complex (Fig. 6*D*).

**Fig. 6:**
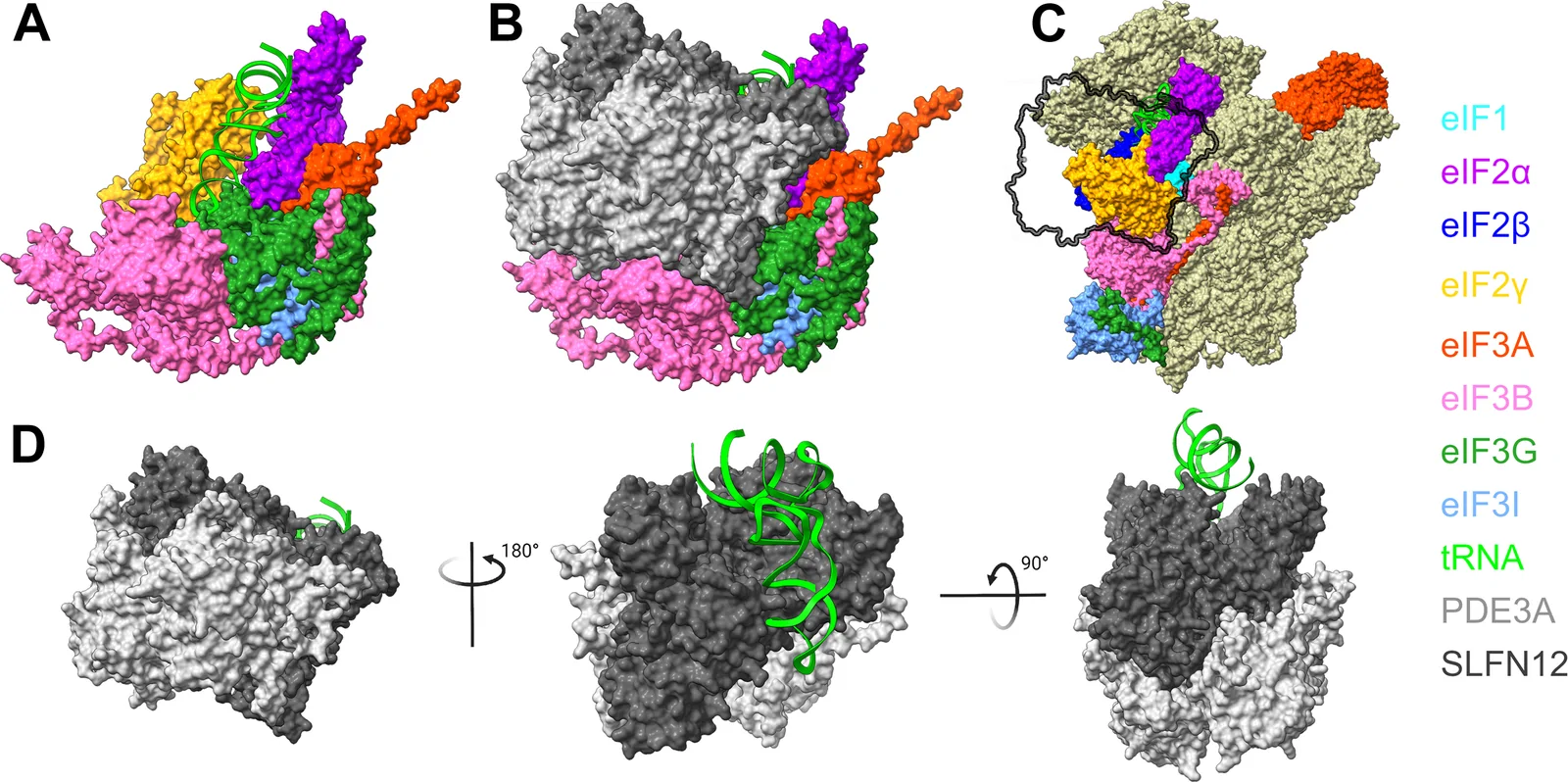
**PDE3A-SLFN12 and partial 43S PIC AlphaFold models.***A*, AlphaFold model of the partial 43S PIC, including eIF2α, eIF2γ, eIF3ACTD, eIF3B, eIF3G, eIF3I and tRNA. *B*, AlphaFold model of a heterotetrameric PDE3A-SLFN12 complex bound to the partial 43S PIC. *C*, Cryo-EM structure of yeast 48S PIC (PDB ID: 6gsm), with the superimposed outline of the AlphaFold-modeled heterotetrameric PDE3A-SLFN12 complex. *D*, orientations of the PDE3A–SLFN12 complex model showing tRNA binding.

The AlphaFold model predicts the PDE3A–SLFN12 complex binding near eIF3B and the heterotrimeric eIF2αβγ-tRNA ternary complex (Fig. 6, *B* and *C*). In this model, one tRNA molecule is bound between the two SLFN12 monomers, with the major cleavage sites of the tRNA in the acceptor stem (21) located close to the active site residues of SLFN12 (Fig. 6*D* and Supplemental Fig. S5). According to the model, the PDE3A–SLFN12 complex interacts with eIF3B and the CTD of eIF3A, but unlikely with eIF3G or eIF3I (Fig. 6*C*), consistent with co-immunoprecipitation and BioID results. Furthermore, eIF2α and eIF2γ interactions were broadly induced in HeLa cells, whereas eIF2β interactions were not detected (Supplemental Table S1), in agreement with the AlphaFold model (Fig. 6, *B* and *C*). Together, the results support the hypothesis that the PDE3A–SLFN12 complex localizes near the mRNA entry channel of the 40S ribosomal subunit and interacts with multiple specific eIF subunits.

## Discussion

Although PDE3A modulators have been characterized with respect to their mechanism of action, pre-clinical efficacy, and even clinical potential (2, 6, 7, 8, 9, 11, 12, 13, 20, 21, 22, 36, 37, 38), the cellular context in which these compounds act has remained incompletely defined. Here, we show the anagrelide-induced PDE3A–SLFN12 complex interacting with the translation initiation machinery in human cancer cell lines and propose a distinct localization for the complex near the mRNA entry channel of the 40S ribosomal subunit. Interactions with the PDE3A–SLFN12 complex were specifically induced with eIF2α, eIF2γ, eIF3A, and eIF3B. This selective interaction pattern suggests that the complex associates temporally with the 43S PIC, rather than the 48S PIC, as interactions with components of the eIF4F complex were consistently suppressed or absent (Supplemental Table S1). Furthermore, the lack of interactions with eIF1A and eIF5, together with the suppression of interactions with octamer core eIF3 subunits, indicates that the association is spatially restricted to the mRNA entry channel, as proposed by the AlphaFold model (Fig. 6), rather than the exit channel or other regions of the pre-initiation complex.

Upon activation, SLFN12 specifically cleaves tRNA^Leu^(TAA), leading to ribosomal stalling at cognate UUA codons, global inhibition of translation, and ultimately, cell death (21). However, ectopic expression of a codon-swapped, SLFN12 RNase-resistant tRNA^Leu^(CAG→TAA) only partially rescues cancer cells from PDE3A modulator-induced cytotoxicity, suggesting that additional mechanisms contribute to their effects. The PDE3A modulator OPB-171775 was later shown to activate the ER stress sensor General Control Nonderepressible 2 (GCN2) and its downstream eIF2α–ATF4–CHOP signaling cascade, possibly in response to ribosomal stalling, significantly contributing to the anticancer activity of OPB-171775^22^. Given that PDE3A is known to form tissue- and even subcellular context–specific signalosome complexes (5), it is likely that PDE3A modulators engage multiple additional cytotoxic pathways beyond SLFN12-mediated RNA cleavage. For example, PDE3A modulators have been reported to induce JNK phosphorylation (21), perturb 14-3-3–PDE3A interactions (7, 9), and sensitize tumor cells to inhibitors of the anti-apoptotic Bcl-2 and Bcl-xL proteins (39). Further characterization of the signaling pathways affected by PDE3A modulation will be critical for defining their full mechanism of action.

Association with the 43S PIC at the mRNA entry channel situates the PDE3A-SLFN12 complex at a key regulatory site during the early stages of translation initiation. The peripheral subunits of eIF3 relocate from the solvent side to the intersubunit side of the 40S ribosomal subunit following mRNA binding and start-codon recognition (transition from 43S PIC to 48S PIC), enabling eIF3B to interact with the eIF2 ternary complex (40, 41). This dynamic rearrangement aligns with the specific interactions we observed (eIF3A, eIF3B, eIF2α, and eIF2γ) and suggests that the PDE3A–SLFN12 complex may sterically hinder the rearrangement of peripheral eIF3 subunits. Recently, perturbations in eIF3 subunit stoichiometry were shown to be often associated with changes in translation of important signaling- and stress-related transcripts such as AKT1, MEK1, ERK1/2, MDM2, and ATF4 (42).

While these findings do not directly address the functional role of the anagrelide-induced PDE3A–SLFN12 complex, defining its temporal and spatial localization within the pre-initiation complex provides important mechanistic context. Our results support a model in which anagrelide reorganizes the PDE3A–SLFN12 interactome rather than simply stabilizing the complex (Fig. 7). Loss of interactions with proteasome subunits and ER protein complexes accompanied the accumulation and cytoplasmic redistribution of PDE3A and SLFN12 that we observed by immunofluorescence and offers a possible basis for the complex stabilization reported previously. The induced interactions centered on the ribosome and the peripheral subunits of eIF3, which together with AlphaFold modeling place the complex at the mRNA entry channel side of the 43S PIC. Whether this positioning functionally impacts translation initiation, and whether the effect differs across PDE3A modulators or target cell types, remains to be determined.

**Fig. 7:**
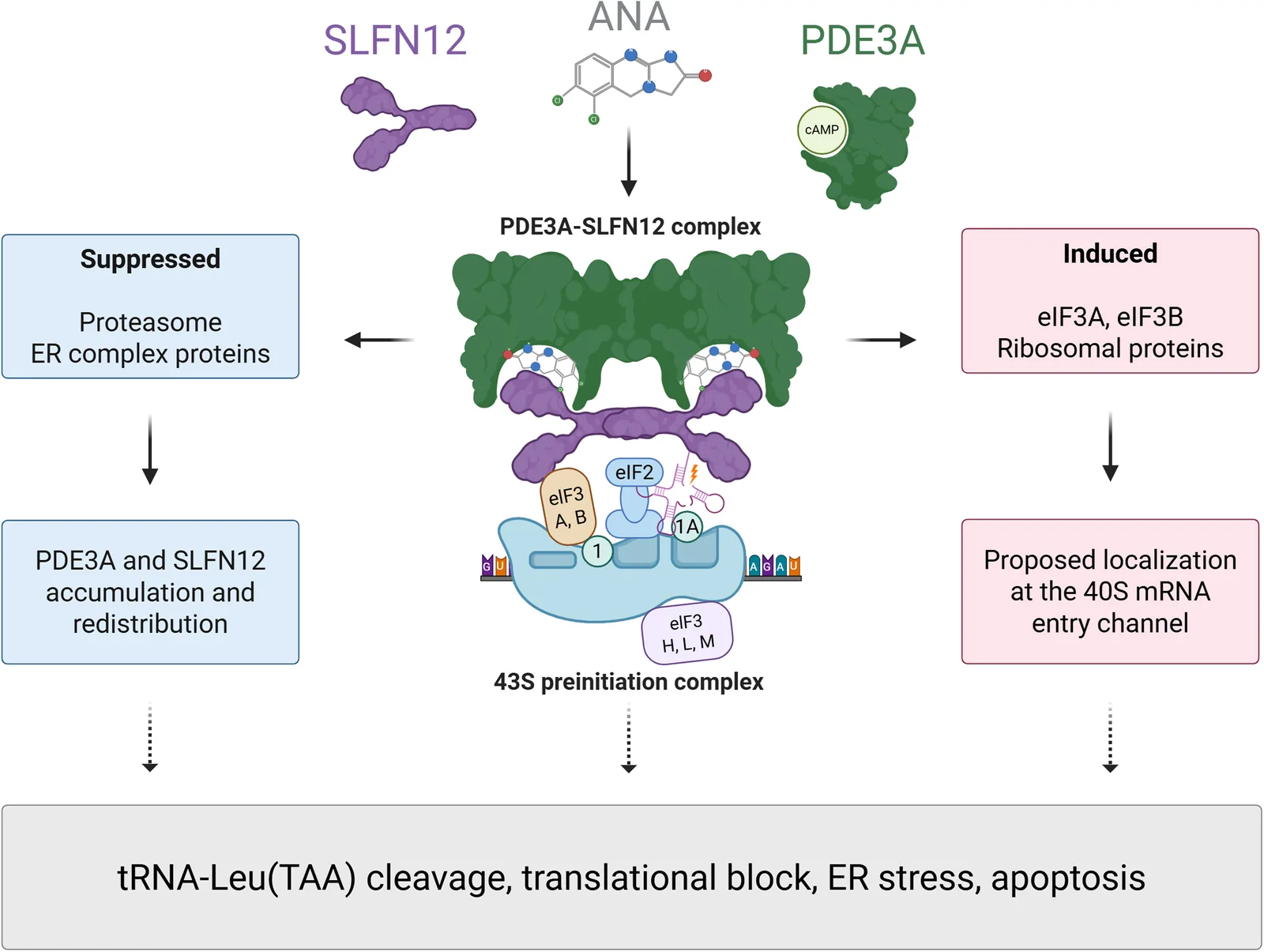
**Model of the anagrelide-induced remodeling of the PDE3A-SLFN12 interactome.** Anagrelide (ANA) promotes formation of the heterotetrameric PDE3A-SLFN12 complex. Interactions suppressed by anagrelide (*left*) included proteasome subunits and ER protein complexes, in line with the accumulation and cytoplasmic redistribution of PDE3A and SLFN12 detected by immunofluorescence. Interactions induced by anagrelide (*right*) included the peripheral eIF3 subunits eIF3A and eIF3B, and combined with AlphaFold modeling, these results support a proposed localization of the complex near the mRNA entry channel of the 40S subunit in the 43S preinitiation complex. *Dotted arrows* indicate downstream events previously reported for the PDE3A-SLFN12 complex, namely tRNA-Leu(TAA) cleavage, translational block, ER stress and apoptosis, which were not assessed in this study. *Created in BioRender*. *Salmikangas*, *S*. (*2026*) https://BioRender.com/nxtrt07.

Interactions with multiple subunits of the regulatory 19S proteasome particle (PSMCs and PSMDs) were suppressed across all bait proteins and in both cell lines (Supplemental Table S1), suggesting that degradation of both PDE3A and SLFN12 via the ubiquitin–proteasome system (UPS) is hindered after anagrelide treatment. Consistently, several additional UPS-related interactions were broadly suppressed, including Ubiquitin Specific Peptidase 14 (USP14), Sequestosome 1 (SQSTM1), and Ubiquitin Protein Ligase E3 Component N-Recognin 4 (UBR4). These findings corroborate earlier observations showing that proteasome inhibition by bortezomib increases ubiquitin-conjugated SLFN12, indicating constitutive UPS degradation of SLFN12 (12). In parallel, interactions with several chaperones and chaperonins, including heat-shock protein families Hsp27, Hsp70, Hsp90, and Hsp110, were sporadically altered (Supplemental Table S1), consistent with prior reports that PDE3A interacts with and is stabilized by both Hsp70 and Hsp90 (43). We showed that both PDE3A and SLFN12 accumulate in cells following anagrelide treatment (Fig. 5*A*), and together these findings suggest that formation of the anagrelide-induced PDE3A–SLFN12 complex is associated with altered protein stabilization, leading to reduced ubiquitination and proteasome-mediated degradation of both proteins.

The PDE3A–SLFN12 complex in our AlphaFold model was predicted with high accuracy and confidence (Supplemental Fig. S4), and although the modeled 43S PIC was only partial due to the AlphaFold Server’s token limit, it provided a biologically plausible fold when compared with the Cryo-EM model of the 48S PIC (Fig. 6, *A* and *C*). Although two separate cryo-EM models of the PDE3A–SLFN12 complex have been reported (6, 36), it has not been resolved in a physiological molecular context—such as in complex with tRNA or with components of the translation initiation machinery. Our predicted model supports the previously hypothesized model (21), where the SLFN12 homodimer clamps one tRNA molecule with the major cleaving sites of the acceptor stem located near the active sites of SLFN12 in the valley between the two monomers (Fig. 6*D* and Supplemental Fig. S5). However, computational predictions alone cannot confirm the precise structural arrangement or binding interfaces, and experimental validation is required to determine the exact structure of these complexes.

The interactomic data were derived only from 2 cell lines, although validated by multiple biological and technical replicates. Further studies should extend analyses to additional cell lines and cancer types and compare a wider panel of PDE3A modulators to clarify how cellular context and modulator-specific interactomes influence cytotoxic responses. Validation of the other BioID-detected interactions beyond the ribosomal partners is essential, as we have only focused on a small subset of the results in this study. Finally, experimentally resolved high-resolution structures of the PDE3A–SLFN12 complex with a bound tRNA complexed by different modulators are needed to validate the computational models.

In this study, we mapped the interactomes for PDE3A, SLFN12, and the anagrelide-induced PDE3A–SLFN12 complex in HeLa cells and SA-4 liposarcoma cells and showed that complex formation promotes relocalization toward the mRNA entry channel of the 40S ribosomal subunit, while inducing interactions with parts of the translation initiation complex. We also observed clear differences in the interactomes between cell lines, highlighting the compartmental and cell-type–specific nature of PDE3A signaling. These findings expand our mechanistic understanding of PDE3A modulators’ effects on associated protein-protein interaction networks and support their continued development as therapeutic agents.

## Data Availability

The computational structure model is available at ModelArchive (https://modelarchive.org/doi/10.5452/ma-20gzt) under accession number 4uxsktFK9S. Mass spectrometry data are available at MassIVE under accession number MSV000100787. Immunofluorescence data are available at BioImage Archive under accession number S-BIAD3037. Other data that support the findings of this study are available from the authors on reasonable request.

## Supplemental Data

This article contains supplemental data.

## Conflict of Interest

The authors declare the following financial interests/personal relationships which may be considered as potential competing interests:

H. S. is a shareholder of and employed by Sartar Therapeutics Ltd. K. T., T. B., and H. S. are inventors in pending patent application WO/2023/105,119 related to the therapeutic use of PDE3A modulators. The authors declare no other competing interests.

## Figures and Tables

**Supplemental Fig. S1:**
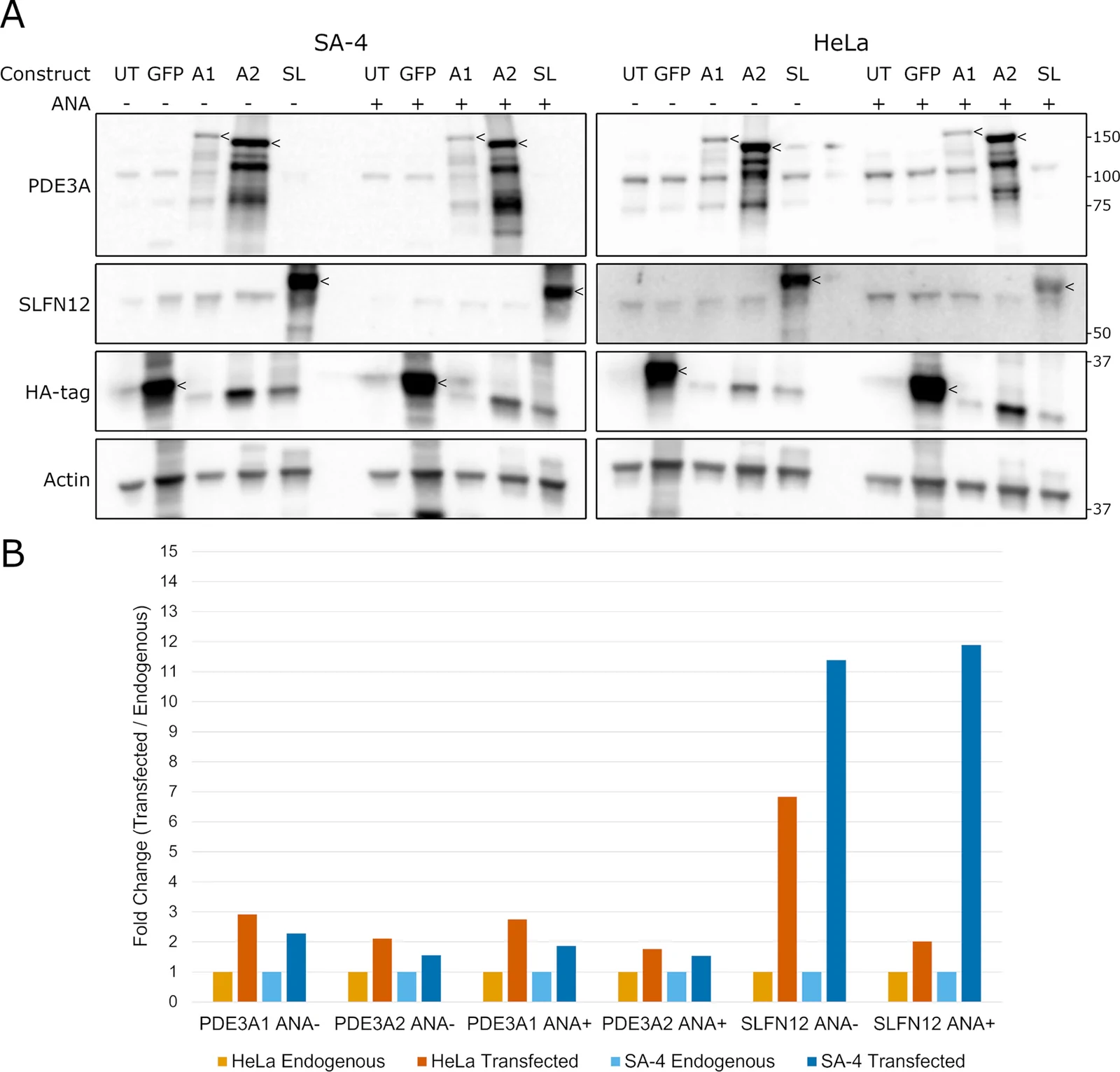
**Expression of the MAC-tagged baits relative to endogenous protein.***A,* immunoblots of whole cell lysates from SA-4 and HeLa cells that were UT or transiently transfected with MAC-GFP (GFP), MAC-PDE3A1 (A1), MAC-PDE3A2 (A2) or MAC-SLFN12 (SL), and treated with DMSO (-) or 100 nM anagrelide (+) for 24 hours. Membranes were probed for PDE3A, SLFN12, the HA epitope of the MAC tag, and actin. Arrowheads mark the MAC-tagged proteins. Molecular weight markers are given in kDa. *B,* densitometric quantification of the blots. For each bait, the tagged band was divided by the endogenous band in the same lane, making the ratio independent of loading, and the endogenous signal was set to 1. MAC-GFP has no endogenous counterpart and was assessed on the HA blot only. ANA, anagrelide; UT, untransfected; PDE3A, phosphodiesterase 3A.

**Supplemental Fig. S2:**
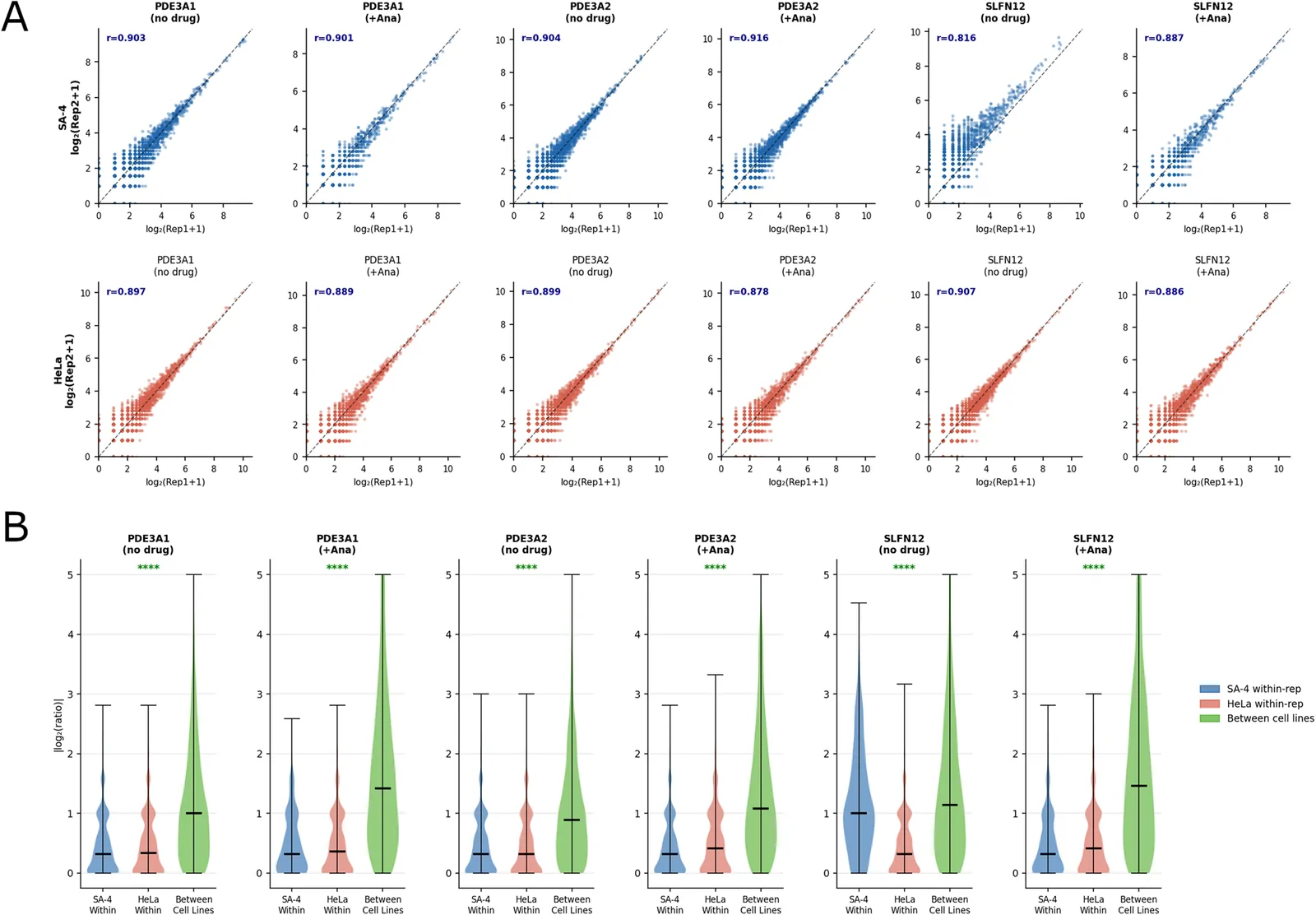
**Reproducibility between biological replicates and comparison with between-cell-line variability.***A,* Log2(x+1)-transformed spectral counts of replicate 1 plotted against replicate 2 for each bait and treatment. Rows show cell line (SA-4, upper; HeLa, lower) and columns the three MAC-tagged baits (PDE3A1, PDE3A2, SLFN12), each with vehicle (no drug) and 100-nM anagrelide (+Ana). Each point represents one identified prey protein and the dashed line indicates identity. Pearson correlation coefficients, calculated on the transformed values, are annotated in each panel. *B,* distributions of absolute log2 ratios for prey proteins shared between cell lines, comparing within-replicate variability in SA-4 (blue) and HeLa (red) with the difference between cell lines for the same bait and treatment (green). Black bars indicate medians, values are capped at 5 for display, and asterisks denote *p* < 0.0001 by 1-sided Mann-Whitney U test (between > within). PDE3A, phosphodiesterase 3A; ANA, anagrelide.

**Supplemental Fig. S3:**
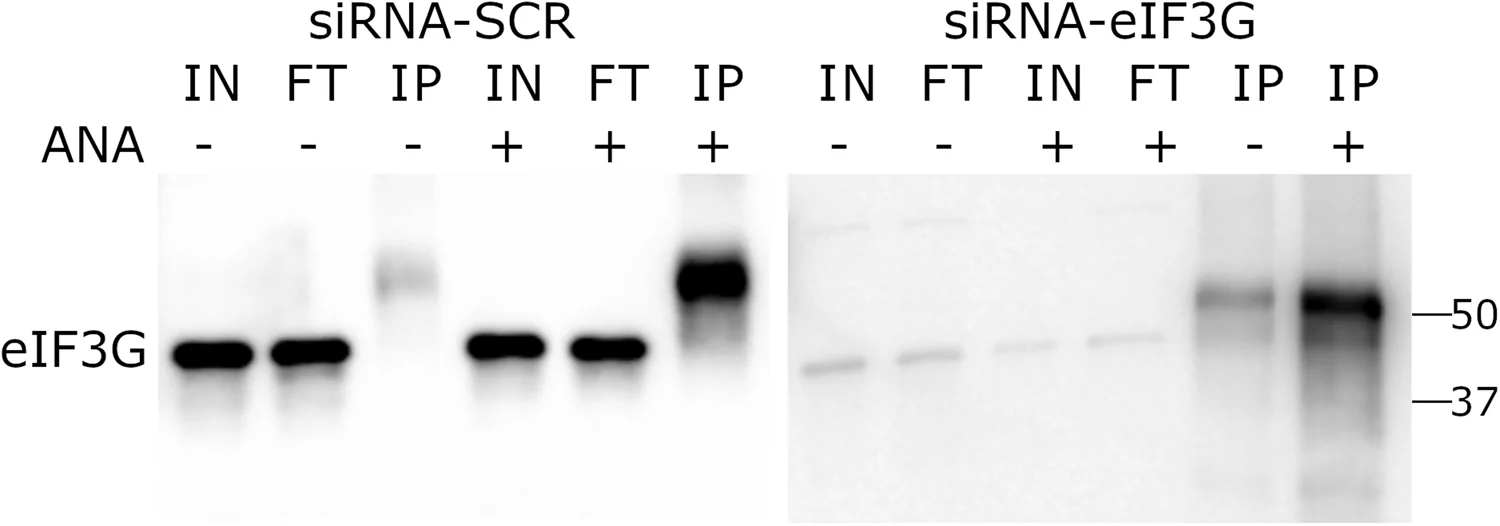
**eIF3G siRNA silencing.** The additional band observed above ∼50 kDa in the eIF3G PDE3A-co-IP samples was confirmed to be nonspecific, as its intensity was unaffected by eIF3G siRNA treatment in the SA-4 cell line. In contrast, the lower molecular weight band (∼<50 kDa), present in the other samples, was efficiently silenced and therefore represents the specific eIF3G signal. PDE3A was used as bait in co-IP. ANA, anagrelide; IN, input; FT, flowthrough sample; IP, immunoprecipitated sample; SCR, nontargeting siRNA; siRNA, Small Interfering RNA; PDE3A, phosphodiesterase 3A; co-IP, co-immunoprecipitation.

**Supplemental Fig. S4:**
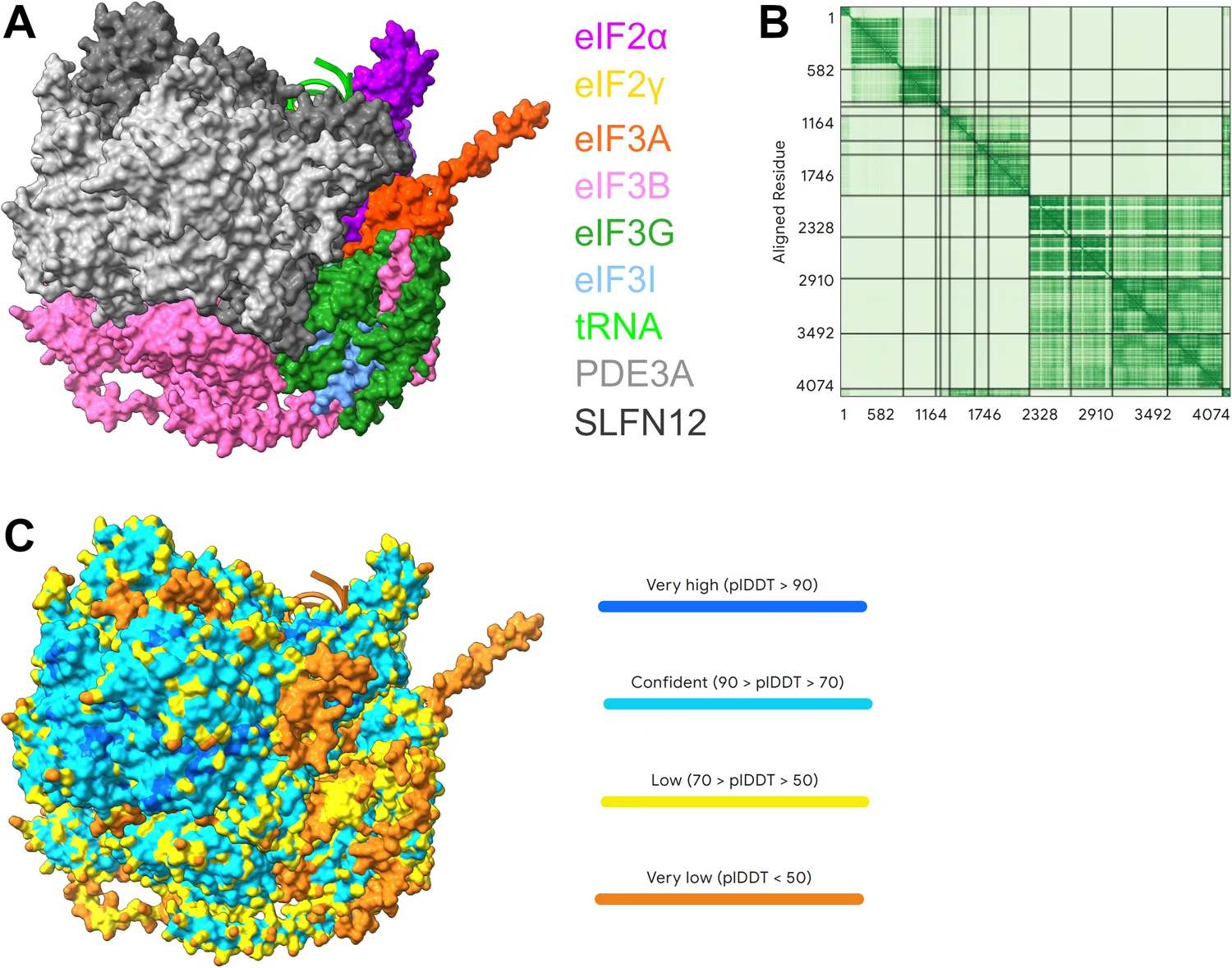
**AlphaFold model confidence estimates.***A,* AlphaFold model of the partial 43S PIC bound to the PDE3A-SLFN12 complex, colored by subunits. *B,* Predicted aligned error (PAE) plot of the AlphaFold model. *C,* The AlphaFold model colored by predicted local distance difference test (pLDDT) per residue. SLFN12, Schlafen family member; PDE3A, phosphodiesterase 3A; PIC, preinitiation complex (43S PIC, 48S PIC); pLDDT, predicted local distance difference test.

**Supplemental Fig. S5:**
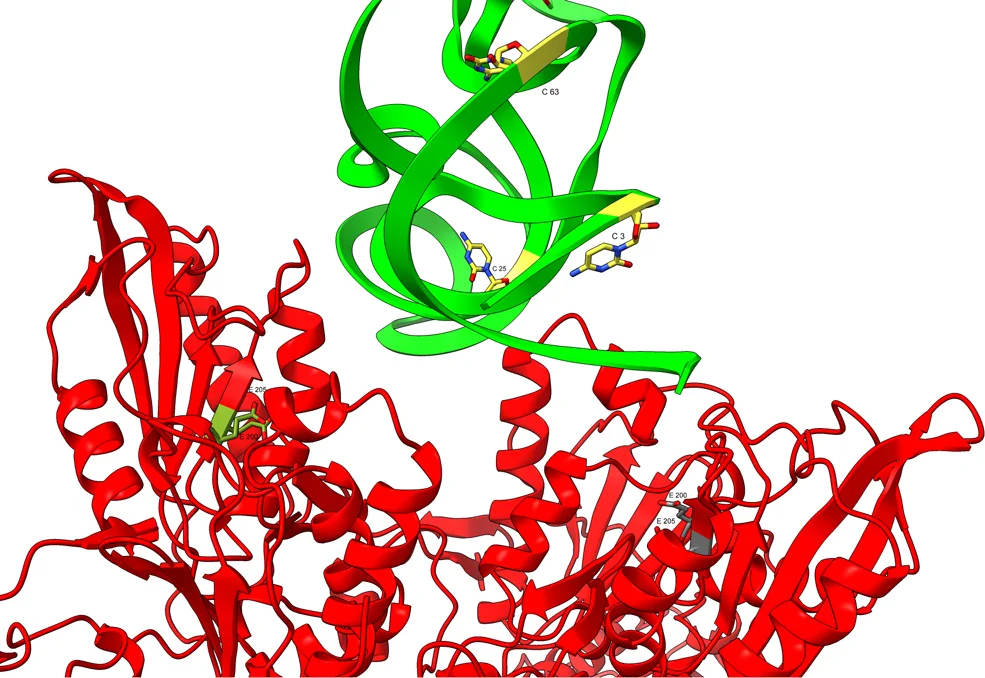
**tRNA cleavage sites and SLFN12 active sites in the AlphaFold Model.** The SLFN12 homodimer (*red*) clamps a single tRNA molecule (*green*). The SLFN12 active site residues and tRNA cleavage sites are presented as sticks and annotated. SLFN12, Schlafen family member.

## Supplemental Data

Supplemental Table S1 High-confidence proximal interactome of PDE3A and SLFN12 and its anagrelide-dependent remodeling. High-confidence proximal interactors of MAC-tagged PDE3A1, PDE3A2, and SLFN12, identified by BioID in SA-4 and HeLa cells treated with anagrelide or vehicle (DMSO), with MAC-GFP as the negative control. Each row represents one bait–prey interaction in one cell line; the table comprises 562 unique high-confidence interactors. Interactions were retained when the prey had an average spectral count ≥10, an average spectral count ≥5% of the corresponding bait's average spectral count, and a BFDR ≤0.05 by SAINT; potential contaminants present in ≥20% of CRAPome control experiments were excluded unless their prey/control spectral-count fold change was ≥3 (“Rescued”). For each interaction the table reports: prey spectral counts per replicate, their sum, average, and number of replicates; GFP-control spectral counts; the SAINT score and BFDR; the prey/GFP spectral-count fold change; UniProt accession, gene and protein names, organism, and protein length; Gene Ontology terms (biological process, cellular component, and molecular function); CRAPome frequency (%) and filter outcome; the prey average spectral count normalized to the bait average spectral count (%); the log2 fold change between anagrelide and vehicle (bait-normalized average spectral counts; positive values indicate increased association upon anagrelide); the resulting change in interaction (NEW, detected only with anagrelide; LOST, detected only with vehicle; UPREGULATED/DOWNREGULATED, increased/decreased with anagrelide); the number of distinct peptides identifying the prey; and the sequence coverage for each prey. Abbreviations: *SAINT*, Significance Analysis of INTeractome; *SLFN12*, Schlafen family member; *PDE3A*, phosphodiesterase 3A; *BFDR*, Bayesian f

Supplemental Table S2 MCL clusters of the anagrelide-modulated interaction networks. Clusters were generated in the STRING database version 12.0 (https://string-db.org/) using MCL with an inflation parameter of 3, applied separately to each of the six subnetworks derived from the 562 high-confidence interactors in Supplementary Table S1. Proteins left unconnected by STRING were not assigned to a cluster. Each sheet holds one network: interactions induced or suppressed by anagrelide in SA-4 cells (*SA-4_Induced*, *SA-4_Suppressed*), in HeLa cells (*HeLa_Induced*, *HeLa_Suppressed*), and in both cell lines (*Both_Induced*, *Both_Suppressed*). Columns give the clustering method, cluster number, the number of genes in the cluster, up to three functional enrichment descriptions returned by STRING (“-” where none was assigned), and the member gene names. Abbreviation: *MCL*, Markov clustering.

Supplemental Table S3 Immunofluorescence quantification of PDE3A and SLFN12 accumulation and colocalization in HeLa and SA-4 cells treated with DMSO or 100-nM anagrelide. *Accumulation results*: per-cell measurements (area, mean intensity, integrated density, raw integrated density, and background-subtracted raw integrated density) for PDE3A and SLFN12, with the mean and SD of the background-subtracted values per condition. *Accumulation Mann-Whitney tests*: two-tailed Mann-Whitney *U* tests comparing anagrelide with DMSO for each protein and cell line. *Colocalization results*: mean Pearson correlation coefficients for SLFN12 vs RPS20, SLFN12 versus PDE3A, and PDE3A vs RPS20 per condition, with the DMSO to anagrelide difference. *Colocalization Pearson R values*: per-cell Pearson R values underlying the summary. *Colocalization Mann-Whitney tests*: two-tailed Mann-Whitney *U* tests comparing anagrelide with DMSO for each protein pair and cell line. Abbreviations: *SLFN12*, Schlafen family member; *PDE3A*, phosphodiesterase 3A.

Supplemental Table S4 Proteins identified in the BioID experiments. All proteins identified in each cell line batch, filtered to below 1% FDR at the peptide-spectrum match, peptide, and protein levels. Sheet 1 lists the 4141 proteins identified in the SA-4 batch and sheet 2 the 3988 proteins identified in the HeLa batch. For each protein the table reports the UniProt accession, gene name, protein length, the number of distinct peptide sequences (combined total peptides, after FDR filtering and razor peptide assignment), sequence coverage (%), the spectral counts in each individual run, and the combined total spectral count across the batch. Abbreviation: *FDR*, false discovery rate.

## References

[1] Baillie G.S., Tejeda G.S., Kelly M.P. (2019). Therapeutic targeting of 3′,5′-cyclic nucleotide phosphodiesterases: inhibition and beyond. Nat. Rev. Drug Discov..

[2] Wu X., Schnitzler G.R., Gao G.F., Diamond B., Baker A.R., Kaplan B. (2020). Mechanistic insights into cancer cell killing through interaction of phosphodiesterase 3A and schlafen family member 12. J. Biol. Chem..

[3] Shakur Y., Takeda K., Kenan Y., Yu Z.X., Rena G., Brandt D. (2000). Membrane localization of cyclic nucleotide phosphodiesterase 3 (PDES) - two N-terminal, domains are required for the efficient targeting to, and association of, PDE3 with endoplasmic reticulum. J. Biol. Chem..

[4] Fu Q., Wang Y., Yan C., Xiang Y.K. (2024). Phosphodiesterase in heart and vessels: from physiology to diseases. Physiol. Rev..

[5] Pati S.R., Sholokh A., Klussmann E. (2025). PDE3A as a therapeutic target for the modulation of compartmentalised cyclic nucleotide-dependent signalling. Cells.

[6] Garvie C.W., Wu X., Papanastasiou M., Lee S., Fuller J., Schnitzler G.R. (2021). Structure of PDE3A-SLFN12 complex reveals requirements for activation of SLFN12 RNase. Nat. Commun..

[7] de Waal L., Lewis T.A., Rees M.G., Tsherniak A., Wu X., Choi P.S. (2016). Identification of cancer-cytotoxic modulators of PDE3A by predictive chemogenomics. Nat. Chem. Biol..

[8] Lewis T.A., de Waal L., Wu X., Youngsaye W., Wengner A., Kopitz C. (2019). Optimization of PDE3A modulators for SLFN12-Dependent cancer cell killing. ACS Med. Chem. Lett..

[9] Pulkka O.P., Gebreyohannes Y.K., Wozniak A., Mpindi J.P., Tynninen O., Icay K. (2019). Anagrelide for gastrointestinal stromal tumor. Clin. Cancer Res..

[10] Sun L., Quan H., Xie C., Wang L., Hu Y., Lou L. (2014). Phosphodiesterase 3/4 inhibitor zardaverine exhibits potent and selective antitumor activity against hepatocellular carcinoma both in vitro and in vivo independently of phosphodiesterase inhibition. PLoS One.

[11] Ai Y., He H., Chen P., Yan B., Zhang W., Ding Z. (2020). An alkaloid initiates phosphodiesterase 3A-schlafen 12 dependent apoptosis without affecting the phosphodiesterase activity. Nat. Commun..

[12] Li D., Chen J., Ai Y., Gu X., Li L., Che D. (2019). Estrogen-related hormones induce apoptosis by stabilizing Schlafen-12 protein turnover. Mol. Cell.

[13] Aquilanti E., Goldoni S., Baker A., Kotynkova K., Andersen S., Bozinov V. (2024). Velcrin molecular glues induce apoptosis in glioblastomas with high PDE3A and SLFN12 expression. Neurooncol. Adv..

[14] Sihto H., Giger O., Toivanen K., Salmikangas S., Vesterinen T., Sampo M. (2025). Phosphodiesterase 3 A expression in gastrointestinal stromal tumors. Virchows Arch..

[15] Toivanen K., Kilpinen S., Ojala K., Merikoski N., Salmikangas S., Sampo M. (2023). PDE3A is a highly expressed therapy target in myxoid liposarcoma. Cancers (Basel).

[16] Jo U., Pommier Y. (2022). Structural, molecular, and functional insights into schlafen proteins. Exp. Mol. Med..

[17] Mavrommatis E., Fish E.N., Platanias L.C. (2013). The schlafen family of proteins and their regulation by interferons. J. Interf. Cytokine Res..

[18] Neumann B., Zhao L., Murphy K., Gonda T.J. (2008). Subcellular localization of the Schlafen protein family. Biochem. Biophs. Res. Commun..

[19] Kobayashi-Ishihara M., Smutná K.F., Alonso F.E., Argilaguet J., Esteve-Codina A., Geiger K. (2023). Schlafen 12 restricts HIV-1 latency reversal by a codon-usage dependent post-transcriptional block in CD4+T cells. Commun. Biol..

[20] Yan B., Ding Z., Zhang W., Cai G., Han H., Ma Y. (2022). Multiple PDE3A modulators act as molecular glues promoting PDE3A-SLFN12 interaction and induce SLFN12 dephosphorylation and cell death. Cell Chem. Biol..

[21] Lee S., Hoyt S., Wu X., Garvie C., McGaunn J., Shekhar M. (2023). Velcrin-induced selective cleavage of tRNA(Leu)(TAA) by SLFN12 causes cancer cell death. Nat. Chem. Biol..

[22] Takaki E.O., Kiyono K., Obuchi Y., Yamauchi T., Watanabe T., Matsumoto H. (2024). A PDE3A-SLFN12 molecular glue exhibits significant antitumor activity in TKI-Resistant gastrointestinal stromal tumors. Clin. Cancer Res..

[23] Roux K.J., Kim D.I., Raida M., Burke B. (2012). A promiscuous biotin ligase fusion protein identifies proximal and interacting proteins in mammalian cells. J. Cell Biol..

[24] Liu X., Salokas K., Tamene F., Jiu Y., Weldatsadik R.G., Ohman T. (2018). An AP-MS- and BioID-compatible MAC-tag enables comprehensive mapping of protein interactions and subcellular localizations. Nat. Commun..

[25] Liu X., Salokas K., Weldatsadik R.G., Gawriyski L., Varjosalo M. (2020). Combined proximity labeling and affinity purification-mass spectrometry workflow for mapping and visualizing protein interaction networks. Nat. Protoc..

[26] Kong A.T., Leprevost F.V., Avtonomov D.M., Mellacheruvu D., Nesvizhskii A.I. (2017). MSFragger: ultrafast and comprehensive peptide identification in mass spectrometry-based proteomics. Nat. Methods.

[27] da Veiga Leprevost F., Haynes S.E., Avtonomov D.M., Chang H.Y., Shanmugam A.K., Mellacheruvu D. (2020). Philosopher: a versatile toolkit for shotgun proteomics data analysis. Nat. Methods.

[28] Teo G.C., Liu G.M., Zhang J.P., Nesvizhskii A.I., Gingras A.C., Choi H. (2014). Improvements and additional features in Significance analysis of INTeractome software. J. Proteomics.

[29] Mellacheruvu D., Wright Z., Couzens A.L., Lambert J.P., St-Denis N.A., Li T. (2013). The CRAPome: a contaminant repository for affinity purification-mass spectrometry data. Nat. Methods.

[30] Szklarczyk D., Nastou K., Koutrouli M., Kirsch R., Mehryary F., Hachilif R. (2025). The STRING database in 2025: protein networks with directionality of regulation. Nucleic Acids Res..

[31] Shannon P., Markiel A., Ozier O., Baliga N.S., Wang J.T., Ramage D. (2003). Cytoscape: a software environment for integrated models of biomolecular interaction networks. Genome Res..

[32] Dunn K.W., Kamocka M.M., McDonald J.H. (2011). A practical guide to evaluating colocalization in biological microscopy. Am. J. Physiol. Cell Physiol..

[33] Abramson J., Adler J., Dunger J., Evans R., Green T., Pritzel A. (2024). Accurate structure prediction of biomolecular interactions with AlphaFold 3. Nature.

[34] Meng E.C., Goddard T.D., Pettersen E.F., Couch G.S., Pearson Z.J., Morris J.H. (2023). UCSF ChimeraX: tools for structure building and analysis. Protein Sci..

[35] Wagner S., Herrmannová A., Sikrová D., Valásek L.S. (2016). Human eIF3b and eIF3a serve as the nucleation core for the assembly of eIF3 into two interconnected modules: the yeast-like core and the octamer. Nucleic Acids Res..

[36] Chen J., Liu N., Huang Y., Wang Y., Sun Y., Wu Q. (2021). Structure of PDE3A-SLFN12 complex and structure-based design for a potent apoptosis inducer of tumor cells. Nat. Commun..

[37] Toivanen K., De Sutter L., Wozniak A., Wyns K., Merikoski N., Salmikangas S. (2025). Pharmacokinetic profile and in vivo anticancer efficacy of anagrelide administered subcutaneously in rodents. Drug Deliv..

[38] Papadopoulos K.P., McKean M., Goldoni S., Genvresse I., Garrido M.F., Li R. (2024). First-in-Human dose-escalation study of the first-in-class PDE3A-SLFN12 complex inducer BAY 2666605 in patients with advanced solid tumors coexpressing SLFN12 and PDE3A. Clin. Cancer Res..

[39] Toivanen K., Murumägi A., Wozniak A., Wyns K., Wang C.-C., De Sutter L. (2025). Phosphodiesterase 3A modulators sensitize tumor cells to Bcl-xL and Bcl-2/Bcl-xL inhibitors. BioRxiv.

[40] Simonetti A., Brito Querido J., Myasnikov A.G., Mancera-Martinez E., Renaud A., Kuhn L. (2016). eIF3 peripheral subunits rearrangement after mRNA binding and start-codon recognition. Mol. Cell.

[41] Llácer J.L., Hussain T., Dong J.S., Villamayor L., Gordiyenko Y., Hinnebusch A.G. (2021). Large-scale movement of eIF3 domains during translation initiation modulate start codon selection. Nucleic Acids Res..

[42] Herrmannová A., Jelínek J., Pospíšilová K., Kerényi F., Vomastek T., Watt K. (2024). Perturbations in eIF3 subunit stoichiometry alter expression of ribosomal proteins and key components of the MAPK signaling pathways. Elife.

[43] Zemskov E.A., Zemskova M.A., Wu X.M., Caceres S.M., Delgado D.C., Yegambaram M. (2024). Novel mechanism of cyclic nucleotide crosstalk mediated by PKG-dependent proteasomal degradation of the Hsp90 client protein phosphodiesterase 3A. J. Biol. Chem..

