# Supplementary material for: Anagrelide Remodels the PDE3A–SLFN12 Interactome to Associate with Translation Initiation Machinery in Cancer Cell Lines
Source: Mol Cell Proteomics. 2026 Sep 18;25(10):101663. doi: 10.1016/j.mcpro.2026.101663 (PMC13693335; doi:10.1016/j.mcpro.2026.101663)
Supplement: Supplementary file 1 [file mmc5.docx]

**Supplementary Table 1. High-confidence proximal interactome of PDE3A and SLFN12 and its anagrelide-dependent remodeling.** High-confidence proximal interactors of MAC-tagged PDE3A1, PDE3A2, and SLFN12, identified by BioID in SA-4 and HeLa cells treated with anagrelide or vehicle (DMSO), with MAC-GFP as the negative control. Each row represents one bait–prey interaction in one cell line; the table comprises 562 unique high-confidence interactors. Interactions were retained when the prey had an average spectral count ≥10, an average spectral count ≥5% of the corresponding bait's average spectral count, and a Bayesian false discovery rate (BFDR) ≤0.05 by Significance Analysis of INTeractome (SAINT); potential contaminants present in ≥20% of CRAPome control experiments were excluded unless their prey/control spectral-count fold change was ≥3 ("Rescued").

For each interaction the table reports: prey spectral counts per replicate, their sum, average, and number of replicates; GFP-control spectral counts; the SAINT score and BFDR; the prey/GFP spectral-count fold change; UniProt accession, gene and protein names, organism, and protein length; Gene Ontology terms (biological process, cellular component, molecular function); CRAPome frequency (%) and filter outcome; the prey average spectral count normalized to the bait average spectral count (%); the log2 fold change between anagrelide and vehicle (bait-normalized average spectral counts; positive values indicate increased association upon anagrelide); the resulting change in interaction (NEW, detected only with anagrelide; LOST, detected only with vehicle; UPREGULATED / DOWNREGULATED, increased / decreased with anagrelide); the number of distinct peptides identifying the prey; and the sequence coverage for each prey.

**Supplementary Table 2. MCL clusters of the anagrelide-modulated interaction networks.** Clusters were generated in the STRING database version 12.0 (<https://string-db.org/>) using Markov clustering (MCL) with an inflation parameter of 3, applied separately to each of the six subnetworks derived from the 562 high-confidence interactors in Supplementary Table 1. Proteins left unconnected by STRING were not assigned to a cluster. Each sheet holds one network: interactions induced or suppressed by anagrelide in SA-4 cells (*SA-4_Induced*, *SA-4_Suppressed*), in HeLa cells (*HeLa_Induced*, *HeLa_Suppressed*), and in both cell lines (*Both_Induced*, *Both_Suppressed*). Columns give the clustering method, cluster number, the number of genes in the cluster, up to three functional enrichment descriptions returned by STRING ("-" where none was assigned), and the member gene names.

**Supplementary Table 3. Immunofluorescence quantification of PDE3A and SLFN12 accumulation and colocalization in HeLa and SA-4 cells treated with DMSO or 100 nM anagrelide.** *Accumulation results*: per-cell measurements (area, mean intensity, integrated density, raw integrated density, and background-subtracted raw integrated density) for PDE3A and SLFN12, with the mean and SD of the background-subtracted values per condition. *Accumulation Mann-Whitney tests*: two-tailed Mann-Whitney U tests comparing anagrelide with DMSO for each protein and cell line. *Colocalization results*: mean Pearson correlation coefficients for SLFN12 vs RPS20, SLFN12 vs PDE3A, and PDE3A vs RPS20 per condition, with the DMSO to anagrelide difference. *Colocalization Pearson R values*: per-cell Pearson R values underlying the summary. *Colocalization Mann-Whitney tests*: two-tailed Mann-Whitney U tests comparing anagrelide with DMSO for each protein pair and cell line.

**Supplementary Table 4. Proteins identified in the BioID experiments.** All proteins identified in each cell line batch, filtered to below 1% false discovery rate at the peptide-spectrum match, peptide, and protein levels. Sheet 1 lists the 4,141 proteins identified in the SA-4 batch and sheet 2 the 3,988 proteins identified in the HeLa batch. For each protein the table reports the UniProt accession, gene name, protein length, the number of distinct peptide sequences (Combined Total Peptides, after FDR filtering and razor peptide assignment), sequence coverage (%), the spectral counts in each individual run, and the combined total spectral count across the batch.

**Supplementary Figure 1. Expression of the MAC-tagged baits relative to endogenous protein.** **A** Immunoblots of whole cell lysates from SA-4 and HeLa cells that were untransfected (UT) or transiently transfected with MAC-GFP (GFP), MAC-PDE3A1 (A1), MAC-PDE3A2 (A2) or MAC-SLFN12 (SL), and treated with DMSO (-) or 100 nM anagrelide (+) for 24 hours. Membranes were probed for PDE3A, SLFN12, the HA epitope of the MAC tag, and actin. Arrowheads mark the MAC-tagged proteins. Molecular weight markers are given in kDa. **B** Densitometric quantification of the blots. For each bait, the tagged band was divided by the endogenous band in the same lane, making the ratio independent of loading, and the endogenous signal was set to 1. MAC-GFP has no endogenous counterpart and was assessed on the HA blot only.

**Supplementary Figure 2. Reproducibility between biological replicates and comparison with between-cell-line variability.** **A** Log2(x+1)-transformed spectral counts of replicate 1 plotted against replicate 2 for each bait and treatment. Rows show cell line (SA-4, upper; HeLa, lower) and columns the three MAC-tagged baits (PDE3A1, PDE3A2, SLFN12), each with vehicle (no drug) and 100 nM anagrelide (+Ana). Each point represents one identified prey protein and the dashed line indicates identity. Pearson correlation coefficients, calculated on the transformed values, are annotated in each panel. **B** Distributions of absolute log2 ratios for prey proteins shared between cell lines, comparing within-replicate variability in SA-4 (blue) and HeLa (red) with the difference between cell lines for the same bait and treatment (green). Black bars indicate medians, values are capped at 5 for display, and asterisks denote p < 0.0001 by one-sided Mann-Whitney U test (between > within).

**Supplementary Figure 3. eIF3G siRNA silencing.** The additional band observed above ~50 kDa in the eIF3G PDE3A-co-IP samples was confirmed to be nonspecific, as its intensity was unaffected by eIF3G siRNA treatment in the SA-4 cell line. In contrast, the lower molecular weight band (~<50 kDa), present in the other samples, was efficiently silenced and therefore represents the specific eIF3G signal. PDE3A was used as bait in co-IP. ANA – anagrelide; IN – input; FT – flowthrough sample; IP – immunoprecipitated sample; SCR – non-targeting siRNA.

**Supplementary Figure 4. AlphaFold model confidence estimates. A** AlphaFold model of the partial 43S PIC bound to the PDE3A-SLFN12 complex, colored by subunits. **B** Predicted aligned error (PAE) plot of the AlphaFold model. **C** The AlphaFold model colored by predicted local distance difference test (pLDDT) per residue.

**Supplementary Figure 5. tRNA cleavage sites and SLFN12 active sites in the AlphaFold Model.** The SLFN12 homodimer (red) clamps a single tRNA molecule (green). The SLFN12 active site residues and tRNA cleavage sites are presented as sticks and annotated.
